# Diets optimized for environmental sustainability and health: implications for diet costs across socio-economic positions for Dutch adults

**DOI:** 10.3389/fnut.2025.1667399

**Published:** 2025-11-17

**Authors:** Reina E. Vellinga, Samantha N. Heerschop, Sander Biesbroek, Pieter van 't Veer, Jose Drijvers, Marieke van Bakel, Anne Hollander, Elisabeth H. M. Temme

**Affiliations:** 1 National Institute for Public Health and the Environment (Netherlands), Bilthoven, Netherlands; 2 Wageningen University & Research, Wageningen, Netherlands

**Keywords:** affordability, diet optimization, sustainable diets, greenhouse gas emissions, diet costs, socio-economic position, diet inequalities, dutch diets

## Abstract

**Background:**

Universal access to healthy, safe, and environmentally sustainable diets is essential across all socio-economic backgrounds to improve human and planetary health.

**Objective:**

This modeling study examined the transition to healthier and more environmentally sustainable diets across socio-economic groups in the Netherlands, and investigated the associated implications for diet costs.

**Methods:**

Food consumption data for 1,747 adults were derived from the Dutch National Food Consumption Survey 2019–2021. Participants were categorized according to their highest attained educational level (low, intermediate, high) as proxy for socio-economic position (SEP). For each individual, the diet was minimized for greenhouse gas (GHG) emissions and maximized for diet quality according to the Dutch Healthy Diet 2015 (DHD15) index. Optimized diets were made using a benchmark approach, involving linear combinations of current diets, either within or across the three educational subgroups. Constraints limited individual dietary changes to within 33% of current consumption, except for less commonly consumed food groups. Diet costs were compared between current and optimized diets. Secondary outcomes included nutritional aspects and additional environmental impact indicators.

**Results:**

The results show that modest dietary changes led to a 19%−24% reduction in GHG emissions and a 52%−56% improvement in diet quality, without increasing median diet costs across socio-economic subgroups. Depending on the educational subgroup, optimized diets included more vegetables, fruits, nuts, legumes, and fish, and less grains, dairy, meat, and sugars. More pronounced improvements were found when the optimization was not stratified by educational level.

**Conclusion:**

Across all socio-economic subgroups, modest dietary adjustments can improve health and environmental sustainability without added costs, offering a viable pathway to bridge socio-economic disparities in diet quality. Furthermore, socio-economic disparities in diet quality can be reduced without additional diet costs, provided these educational subgroups are willing and facilitated to adopt diets divergent from their peer group.

## Introduction

Food production and consumption significantly contribute to global environmental impact and health burden (1, 2). Globally, current food systems are responsible for up to 30% of greenhouse gas (GHG) emissions, 70% of freshwater use, and significant land occupation, deforestation, and biodiversity loss (1) Animal-based foods, such as meat and dairy, are generally the main dietary contributors to environmental impacts (3, 4). At the same time, unhealthy diets contribute to a double burden of undernutrition and rising rates of obesity and non-communicable diseases (NCDs) worldwide (5, 6). Transitioning to more plant-based and less animal-based diets is recognized as key to improving both environmental sustainability and public health.

In the Netherlands, animal-based foods such as meat and dairy are with approximately 50% the main contributors to dietary environmental impact, while meat consumption remains above recommendations and intake of fruits, vegetables, and legumes, associated with lower environmental impacts and reduced risk of non-communicable diseases (NCDs), is insufficient (7, 8). Reducing meat and increasing plant-based foods, is key to improving public and environmental health (1). To achieve healthy, sustainable diets, the Dutch government has set targets for an animal to plant protein intake of 50/50 in 2030 (9), however the Dutch ratio of animal to plant protein intake has remained unchanged at 60/40 since 1987 (10–12).

Food costs are of major importance as facilitating or inhibiting factors of dietary choices (13), but often neglected in nutritional research. Generally, healthier foods such as fresh vegetables and fruits are more expensive compared to less healthy, energy-dense foods, for example foods high in sugar, fat, and sodium (14, 15), which may hinder transitioning to affordable, healthy, and sustainable diets (16). Additionally, the rising food prices resulting from both inflationary pressures and population growth-induced scarcity have become an area of significant concern and pose a major challenge for adopting healthy and environmentally sustainable diet that is affordable for all (16, 17). This challenge arises as policy goals strive for universal access to affordable healthy and sustainable dietary patterns (9). Individuals with lower socio-economic positions (SEP) may face greater barriers to accessing healthy diets (18, 19), which widens disparities in diet quality, health outcomes, and related healthcare costs (20, 21). These socio-economic disparities point out the key role of food costs in shaping divergent dietary patterns among individuals from socio-economic subgroups.

Mathematical optimization models have been increasingly used to design diets that aim to balance health and environmental objectives. These models often face challenges, such as trade-offs between sustainability, health, and affordability (22–24). A benchmarking approach developed by Kanellopoulos et al., optimizes diets as linear combinations of current diets of peers (25). Consequently, the modeled diets closely align with existing food consumption patterns, as demonstrated by previous studies based on this model (26, 27). While earlier modeling studies of (sustainable) diets not often included economic aspects such as cost and affordability, these factors are fortunately now being considered more frequently alongside environmental outcomes and diet quality. However, unlike previous studies, we simultaneously optimize for the environmental sustainability, diet quality, taking into account affordability and socio-economic disparities.

As universal access to healthy, safe, environmentally sustainable, and affordable diets is imperative for individuals across all socio-economic positions, it is important to include both costs and socioeconomic positions in analyses. Therefore, the aim of this study is to examine the transition to healthier and more environmentally sustainable diets across socio-economic groups in the Netherlands, and to investigate the associated implications for diet costs. By optimizing diets on environmental sustainability and diet quality, we explore how these changes affect diets and costs among different socio-economic groups.

## Methods

### Study population

Data were derived from the Dutch National Food Consumption Survey 2019–2021, designed to gain insight into diets of children and adults living in the Netherlands (12). Dutch children and adults aged 1–79 years were drawn from a consumer panel and form a representative sample based on age, sex, education, region, and level of urbanization. At baseline, participants completed a digital questionnaire which provided information on height, weight and education. Information on body composition was gathered in different ways depending on age. Height was not measured for adults aged 71–79 years due to practical reasons. Body Mass Index (BMI) was calculated as the average body weight (in kg) divided by average height (in m) squared (kg/m^2^) and categorized as underweight or normal weight (< 25 kg/m^2^) and overweight or obese (>25 kg/m^2^). For this study, 1,747 Dutch adults aged 18–79 years were included.

### Dietary assessment and food composition

Dietary intake was assessed through two non-consecutive 24-h recalls conducted by trained dietitians via telephone or face-to-face interviews, using the GloboDiet system. During each recall, participants were asked to report all foods and beverages consumed during the previous day, including breakfast, lunch, dinner, and any snacks, as applicable. The period between the two 24-h dietary recalls was about 4 weeks. Nutritional intake was estimated with the Dutch Food Composition database (NEVO-online version 2021/7) (28).

### Assessment of socio-economic position

Socio-economic position is a multifaceted concept, often captured by three proxy measures: education, occupation, and income (29). In this study, socio-economic position (SEP) was determined using educational level as a proxy, as income data were not available. Participants' highest attained educational level was obtained from the consumer panel, where this information was self-reported upon recruitment. Education level was categorized into three groups: low (primary education, lower vocational education, or elementary education), intermediate (intermediate vocational education or higher secondary education), and high (higher vocational education or university degree).

### Assessment of dietary costs

Individual food and diet costs were derived using established methods (30). Dutch food retail prices were linked to individual food consumption data from DNFCS 2019–2021. Food prices at barcode level were collected by Questionmark Intelligence B.V. using web-scraping techniques (*n* = 32,135 products). This technique depends on webpage content and therefore data were cross-checked with the Dutch branded food database (31). Food prices at barcode level from 1 Jan 2020 to 1 Jan 2021 were derived from seven supermarket chains. Data were collected every 2 weeks to take into account seasonality. Price promotions were reported but not included. For all barcodes the lowest retail prices were preferable. The barcodes were accordingly categorized as generic food product, and therefore the prices of individual barcodes were summarized as minimal (lowest), mean and median food prices. Outliers were removed by excluding food prices above and below 1.5 times the interquartile range based on food product level. The generic food prices per food product level were linked to generic food items of NEVO database (28), or home-made dishes and composite dishes, ingredients were separately reported in DNFCS, and prices were linked at ingredient level. In case recipes were available from NEVO, these recipes were used. For example, for “fruit citrus average,” the underlaying citrus fruits were attributed based on NEVO recipes (28). All prices were adjusted for preparation and waste and were expressed in euros per edible portion. The variable obtained for each participant was the minimum costs value of their diet in euros per day. Sensitivity analyses were performed using instead of the minimum costs of the aggregated food prices, the mean and median costs of the aggregated food prices.

### Assessment of environmental sustainability

The environmental impact of food consumption was evaluated for greenhouse gas (GHG) emissions (kg CO_2_-eq), land use (m^2^^*^ year), blue water use (also known as irrigation water) (m^3^), acidification (kg SO_2_-eq), fresh water eutrophication (kg P-eq), and marine eutrophication (kg N-eq). Data were derived from the Dutch Life cycle analysis (LCA) Food database version 2021 (32). The environmental impacts were based on Life cycle analysis (LCA) methodology, which quantified the environmental impact through the foods' entire life cycle, from cradle to waste. The LCAs performed had an attributional approach and hierarchical perspective and were performed following the ISO 14040 and 14044 guidelines. GHG emissions were recalculated following the guidelines of the Intergovernmental Panel on Climate Change (IPCC; 2006) (33). Economic allocation was applied when production processes led to more than one food product, except for milk, for which bio-physical allocation was used. The functional unit used was 1 kg of prepared food or drink on the plate. The LCA food database provided primary data for approximately 250 foods and drinks, which cover 75% of food consumption in the Netherlands (7, 32, 34). Foods and beverages for which primary data were not available were matched to similar foods, according to established methods (7, 34). Foods were matched by expert judgement of a panel of scientists and were based on similarities in types of food, production systems and ingredient composition. For composite dishes, standardized recipes from the Dutch Food composition table (NEVO-online version 2021/7) (28) were used where available and if not available, recipes were based on label information. More detailed information on the use of the database can be found elsewhere (7, 32, 34).

### Assessment of healthiness of diet

The healthiness of the diets was assessed by means of the Dutch Healthy Diet 2015 index (DHD15) (35), which measures adherence to the Dutch dietary guidelines. The method scores dietary intake (0–10 points per component) for vegetables, fruits, wholegrain products, legumes, nuts, dairy, fish, tea, fats and oils, coffee, red meat, processed meat, sweetened beverages and fruit juices, alcohol, and sodium, based on average intake over 2 days. Intakes between minimum and maximum values were scored proportionally, and all components were equally weighted and summed. Coffee was excluded due to unavailable data on consumption type (e.g., filtered or unfiltered). The total DHD15 score ranged from 0 (minimal adherence) to 140 (maximal adherence).

### Data-analysis

To identify more environmentally sustainable, healthier, and acceptable diets, the SHARP (Sustainable, Healthy, Affordable, Reliable and Preferrable) model was applied (25–27). This model generates improved diets for each individual person within a population, guided by predefined objectives and constraints. The optimization makes linear combinations of current diets that are benchmarked for higher DHD15 index and lower GHG emissions than the index person. Using linear combinations of the diets of peers (e.g., in this case the subgroups according to educational level) within the population eliminates the need to formulate explicit expert-based acceptability constraints. Consequently, the optimized diets do not surpass the best dietary options present within the target population, and stay within the range of options that are acceptable in the cultural food context of the population.

For the optimization, first the food consumption, GHG emissions, and DHD15 components were calculated based on individual dietary intake data, representing the current diet. Secondly, the current diets were optimized while adhering to a set of constraints. The primary objective was to minimize GHG emissions, and improve DHD15 index, which resulted in trade-offs between minimizing GHG emissions and maximizing the DHD15 index (Supplemental file S1). Each individual's diet was optimized in five models, in which GHG emissions was minimized, while a constraint on the minimum DHD15 index was increased gradually (Supplemental file S1). The difference of the DHD15 index between the first (model 1) and the fifth optimization (model 5) was assessed for each individual. The lower bound of the DHD15 index was defined by minimizing GHG emissions, setting this minimum GHG emissions as maximum constraint and consequently maximizing the DHD15 index. The upper bound was defined by maximizing the DHD15 index.

The five models were run separately for the three levels of SEP and, in a secondary analysis, the five models were run without stratifying for SEP. All optimizations were subject to the following additional constraints: (1) the value/score of the individual components of DHD15 index could not be lower than the values/scores of the observed diet (e.g., diet had to be healthier/maximizing DHD15 index); (2) energy and protein intake were set to be within ±5% of the current intake to maintain current energy and protein levels rather than substantially altering them, and (3) acceptability constraints were set with a range of ±33% based on individuals' food group intake, except when the intake was 0. A difference of 33% was chosen as this is arbitrarily considered an acceptable mean change. For zero intakes, food consumption generally should increase and therefore no constraints were set (e.g., legumes, nuts and fish).

For presentation purposes, food consumption for the optimized diets was aggregated in (sub)food groups. Primary outcomes were GHG emissions, DHD15 index, and diet costs. Secondary outcomes include energy and protein intake, proportion of plant protein, environmental impact indicators: land use, eutrophication, acidification and blue water consumption, food consumption according to food groups, and the nutrients: sodium, vitamin A, B2, B6, B12, calcium, iron, iodine, zinc, and EPA/DHA.

Results are displayed as numbers or proportions, mean (standard deviation, SD) or median (25th−75th percentile) where appropriate. Characteristics of the population and their current diets were summarized for the total population and stratified by level of education. Differences in participants baseline characteristics and diets were assessed for trend using a Cochran-Armitage test for categorical data, regression for parametric continuous data and, Jonckheere-Terpstra test for non-parametric continuous data. All reported *P*-values are two-tailed, and *P* < 0.05 was considered significant. SAS statistical software version 9.4 (SAS Institute Inc., Cary, NC, USA) was used to estimate current diets and its characteristics, and to summarize outcomes of the optimization. FICO Xpress version X was used to determine the optimized diets. Outcomes from the five models were summarized using descriptive statistics. The primary outcomes GHG emissions, DHD15 index, and diet costs were plotted for each individual and summarized, for the current diets and five models. Relative differences in diet costs between the current and optimized diets were summarized using descriptive statistics and visualized by a graph. Food consumption and secondary outcomes for the optimized diet were summarized using descriptive statistics.

## Results

### Population and dietary characteristics

Of the 1,747 Dutch participants aged 18–79 years, 50% was female (Table 1) with a mean age of 55 y (SD = 15) and BMI of 26.7 kg/m^2^ (SD = 5.1). Among the participants, 25% had a low educational level (LEL), 36% had an intermediate educational level (IEL), and 38% had a high educational level (HEL). Participants with LEL were more likely to be female (56%), of higher age (60.8 years), and had a higher BMI (27.7 kg/m^2^), compared to participants with IEL and HEL. The proportion of overweight or obesity was 69% among participants with LEL, and respectively 60 and 47% among IEL and HEL.

**Table 1:** Population and dietary characteristics for 1,747 Dutch adults aged 18–79 years with low, intermediate and high educational level, from the DNFCS 2019–2021.

	**Total** ***n*** = **1,747**		**Low educational level**		**Intermediate educational level**		**High educational level**		
			***n*** = **445**	**(25%)**	***n*** = **633**	**(36%)**	***n*** = **669**	**(38%)**	***P*** **-trend**
**Demographics**									
Sex									< 0.001
Females	867	(50%)	250	(56%)	317	(50%)	300	(45%)	
Males	880	(50%)	195	(44%)	316	(50%)	369	(55%)	
Age	55.0	(15.49)	60.8	(14.61)	52.5	(15.53)	53.4	(15.04)	< 0.001
BMI (kg/m^2^)	26.6	(5.08)	27.7	(5.02)	27.1	(5.61)	25.5	(4.31)	< 0.001
Weight status									
Normal weight (BMI < 25 kg/m^2^)	746	(43%)	138	(31%)	253	(40%)	355	(53%)	< 0.001
Overweight and obese (BMI ≥ 25 kg/m^2^)	1,001	(57%)	307	(69%)	380	(60%)	314	(47%)	
**Diet expenses**									
Diet costs (€/day)	3.31	(2.56–4.26)	3.15	(2.43–4.02)	3.26	(2.56–4.17)	3.48	(2.67–4.58)	< 0.001
**Dietary pattern**									
Diet quality (DHD15 index)	74	(62–86)	70	(59–84)	73	(62–84)	77	(66–89)	< 0.001
Energy (kcal)	2,059	(608)	1,977	(565)	2,061	(619)	2,111	(619)	< 0.001
Protein (g)	81	(25)	80	(24)	81	(27)	81	(24)	0.61
Protein (En%)	16	(4)	16	(4)	16	(3)	16	(3)	< 0.001
Ratio plant/animal protein	40/60		36/64		38/62		42/58		< 0.001
**Environmental impact**									
GHG emissions (kg CO_2_-eq/day)	4.77	(3.87–5.93)	4.80	(3.91–6.01)	4.78	(3.89–5.87)	4.72	(3.84–5.96)	0.55
Land use (m^2*^year/day)	2.92	(2.35–3.55)	2.93	(2.37–3.58)	2.92	(2.35–3.56)	2.88	(2.37–3.47)	0.63
Freshwater eutrophication (g P-eq/day)	0.35	(0.28–0.43)	0.34	(0.28–0.42)	0.35	(0.29–0.43)	0.35	(0.28–0.43)	0.22
Marine water eutrophication (g N-eq/day)	7.34	(5.51–10.01)	7.73	(5.74–10.34)	7.44	(5.5–9.99)	7.12	(5.43–9.46)	< 0.001
Acidification (g SO_2_-eq/day)	43.92	(32.63–60)	46.17	(34.4–63.21)	44.20	(32.75–60.05)	41.83	(31.55–57.5)	< 0.001
Blue water (m^3^/day)	0.15	(0.11–0.21)	0.13	(0.1–0.18)	0.15	(0.11–0.21)	0.17	(0.12–0.24)	< 0.001

Values are presented as proportions, means (SD) and medians (25th−75th percentile).

DHD15; Dutch Healthy Diet 2015 index.

Diets of participants with LEL had, compared to IEL and HEL, a lower DHD15 index (respectively 70, 73, and 77 points), contained on average less energy (respectively 1,977, 2,061, and 2,111 kcal), and had a lower plant protein proportion (respectively 36%, 38%, and 42%; Table 1). The median (25th−75th percentile) daily diet costs were lower for participants with LEL (€3.15 (€2.43–4.02)) than for those with IEL (€3.26 (€2.56–4.17)), and HEL (€3.48 (€2.67–4.58)). The median dietary environmental impacts of participants with LEL were comparable to those of IEL and HEL, except for usage of blue water; which was lower, with respectively 0.13, 0.15, and 0.17 m^3^ per day.

Participants with LEL consumed lower amounts of vegetables (−47 g, −15%), grains and grain-based products (−8 g, −54%), fruits and olives (−34 g, −21%), meat substitutes (−4 g, −62%) and dairy substitutes (−12 g, −122%), nuts and seeds (−7 g, −39%), cheese (−7 g, −17%), fish (−5 g, −25%), water (−155 g, −19%), juices (−8 g, −23%), and alcoholic beverages (−27 g, −20%) compared to those with HEL (Table 2). Participants with LEL consumed more dairy (+49 g, +17%), meat (+27 g, +34%), potatoes (+12 g, +19%), and soft drinks (+35 g, +21%) than those with HEL. The consumption of legumes, eggs, fats and oils, sugar and confectionery, biscuits and pastries, condiments and sauces, soups and bouillon, and savory snacks were approximately similar (± 5 g/day) across the subgroups.

**Table 2:** Mean food intake for current diet (grams per day and %), for 1,747 Dutch adults aged 18–79 years with low, intermediate and high educational level, from the DNFCS 2019–2021.

	**Total (*****n*** = **1,747)**		**Low educational level (*****n*** = **445)**		**Intermediate educational level (*****n*** = **633)**		**High educational level (*****n*** = **669)**		
	**Gram**	**%**	**Gram**	**%**	**Gram**	**%**	**Gram**	**%**	***P*** **-trend** ^a^
**Plant-based food groups**									
Grains and grain-based products	186	5.6%	167	5.3%	188	5.6%	196	5.7%	0.03
Vegetables	171	5.1%	150	4.7%	157	4.7%	197	5.7%	< 0.001
Fruit and olives	145	4.3%	131	4.1%	135	4.0%	165	4.8%	< 0.001
Potatoes	70	2.1%	76	2.4%	72	2.1%	64	1.9%	< 0.001
Nuts and seeds	15	0.5%	11	0.4%	15	0.4%	18	0.5%	< 0.001
Legumes	7	0.2%	7	0.2%	7	0.2%	8	0.2%	0.90
**Animal-based food groups**									
Dairy	314	9.4%	347	11.0%	309	9.2%	297	8.6%	< 0.001
Meat	91	2.7%	104	3.3%	95	2.8%	78	2.3%	0.03
Cheese	36	1.1%	32	1.0%	35	1.0%	39	1.1%	< 0.001
Fish	18	0.5%	15	0.5%	17	0.5%	20	0.6%	0.08
Eggs	17	0.5%	16	0.5%	18	0.5%	16	0.5%	0.30
Dairy replacers	11	0.3%	6	0.2%	12	0.4%	14	0.4%	0.01
Meat replacers	5	0.1%	3	0.1%	3	0.1%	7	0.2%	< 0.001
**Mixed food groups**									
Soup and bouillon	22	0.6%	22	0.7%	18	0.5%	25	0.7%	0.31
Biscuits and pastries	40	1.2%	42	1.3%	38	1.1%	40	1.2%	0.10
Condiments and sauces	35	1.1%	34	1.1%	35	1.0%	36	1.1%	0.62
Sugar and confectionary	23	0.7%	21	0.7%	24	0.7%	24	0.7%	0.90
Fats and oils	23	0.7%	23	0.7%	22	0.7%	22	0.7%	0.04
Savory snacks	16	0.5%	12	0.4%	19	0.6%	16	0.5%	0.42
Miscellaneous	2	0.1%	1	0.0%	3	0.1%	3	0.1%	0.31
**Beverages**									
Coffee and tea	898	26.8%	898	28.4%	852	25.3%	940	27.4%	0.60
Water	855	25.6%	713	22.5%	927	27.5%	881	25.6%	0.03
Soft drinks	191	5.7%	199	6.3%	212	6.3%	164	4.8%	0.02
Fruit and vegetable juice	33	1.0%	26	0.8%	37	1.1%	34	1.0%	0.18
Alcohol beverages	121	3.6%	106	3.3%	120	3.6%	132	3.8%	0.05

^a^p-trend is for %.

Participants with LEL allocated a relatively higher proportion of their expenditure to animal-based food, in contrast to those with IEL and HEL (respectively 43%, 41%, and 37%; Table 3). Additionally, they allocated a relatively lower proportion of their spending to plant-based food (respectively, 32%, 34%, and 38%).

**Table 3:** Average diet expenses (in %) of the current diet per food group for 1,747 Dutch adults aged 18–79 years with low, intermediate and high educational level, from the DNFCS 2019–2021.

	**Total (*n* = 1,747)**	**Low educational level (*n* = 445)**	**Intermediate educational level (*n* = 633)**	**High educational level (*n* = 669)**	***P*-trend**
**Plant-based food groups**					
Grains and grain-based products	7.2%	6.9%	7.4%	7.3%	0.07
Vegetables	12.9%	11.6%	12.1%	14.4%	< 0.001
Fruit and olives	10.5%	9.3%	10.0%	11.7%	< 0.001
Potatoes	2.1%	2.2%	2.1%	1.9%	0.004
Nuts and seeds	2.2%	1.7%	2.2%	2.6%	< 0.001
Legumes	0.2%	0.2%	0.2%	0.3%	0.67
**Animal-based food groups**					
Dairy	8.6%	10.0%	8.5%	7.7%	< 0.001
Meat	18.8%	22.4%	19.7%	15.6%	< 0.001
Cheese	6.4%	6.0%	6.3%	6.8%	0.01
Fish	4.2%	3.6%	4.0%	4.7%	0.02
Eggs	1.5%	1.6%	1.7%	1.4%	0.16
Dairy replacers	0.4%	0.2%	0.4%	0.4%	0.04
Meat replacers	0.8%	0.5%	0.6%	1.3%	< 0.001
**Mixed food groups**					
Soup and bouillon	0.2%	0.2%	0.2%	0.2%	0.54
Biscuits and pastries	2.6%	2.9%	2.5%	2.5%	0.09
Condiments and sauces	2.2%	2.0%	2.2%	2.4%	0.05
Sugar and confectionary	1.9%	1.6%	2.0%	2.0%	0.06
Fats and oils	1.6%	1.6%	1.4%	1.7%	0.22
Savory snacks	2.0%	1.6%	2.4%	1.8%	0.72
Miscellaneous	0.3%	0.1%	0.4%	0.3%	0.49
**Beverages**					
Coffee and tea	4.6%	5.3%	4.6%	4.0%	< 0.001
Water	0.6%	0.6%	0.7%	0.6%	0.68
Soft drinks	2.8%	3.2%	2.9%	2.4%	0.01
Fruit and vegetable juice	1.1%	0.9%	1.1%	1.2%	0.24
Alcohol beverages	4.4%	3.8%	4.2%	4.9%	0.006

### Diet costs of optimizing diets for GHG emissions and Dutch Healthy Diet 2015 index

**Figure 1:**
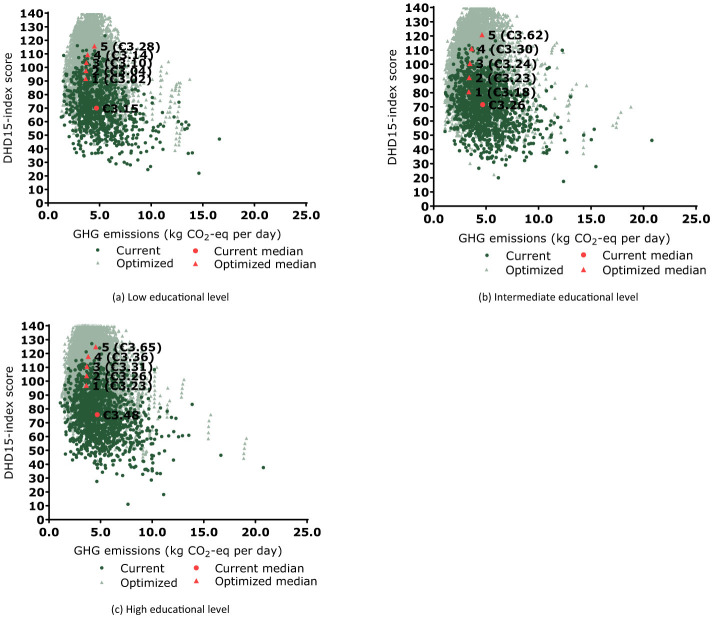
Trade-off between minimizing greenhouse gas (GHG) emissions and maximizing Dutch Healthy Diet index 2015 for 1,747 Dutch adults aged 18–79 years with low **(a)**, intermediate **(b)** and high **(c)** educational levels, from the DNFCS 2019–2021. Values are presented as medians. Dark green circles represent the individual data of current diets and the light green triangles for the optimized diets for all five models 1–5. The circle indicates the median of current diets, the triangles numbered 1–5, represent the median of the models, with median prices. Supplemental file S2 contains the descriptive data.

For participants with LEL, optimizing current diets for GHG emissions while improving the DHD15 index in model 1 (prioritizing GHG emissions) resulted in a reduction of 24% in GHG emissions to 3.65 kg CO_2_-eq and an increase of 30% in the DHD15 index to 92 points compared to current diets (Figure 1a). This diet had also the lowest costs (€3.02). The fifth model (prioritizing diet quality) had the highest GHG emissions of the modeled diets, but also the highest DHD15 index, with 4.49 kg CO_2_-eq (−6%) and 116 points (66%), respectively. This diet was also the most expensive of all diets (€ 3.28). The less stringent models 2–4 showed lower benefits for GHG emissions while the DHD15 index increased, and were less expensive than current diets. The fourth model was selected as the preferable model, as it balanced GHG emissions and DHD15. This preferable model showed a 20% reduction in GHG emissions to 3.83 kg CO_2_-eq and a 56% increase in the DHD15 index to 109 points. Diet costs of the preferable model were €3.14, and were approximately similar to costs of current diets (€3.15). Similar trends were found for preferable diets for IEL and HEL. For participants with IEL, the preferable model showed a 24% reduction in GHG emissions to 3.64 kg CO_2_-eq and a 52% increase in the DHD15 index to 111 points compared to current diets (Figure 1b). Diet costs were with €3.30 approximately similar to costs of current diets (€3.26). For participants with HEL, the preferable model showed a 19% reduction in GHG emissions to 3.81 kg CO_2_-eq and a 53% increase in the DHD15 index to 118 points compared to current diets (Figure 1c). Diet costs for the preferable diet were with €3.36 slightly lower than costs of current diets (€3.48).

**Figure 2:**
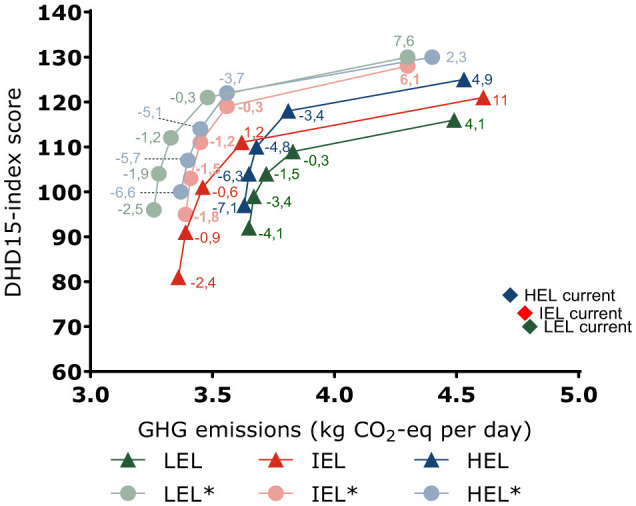
Average greenhouse gas (GHG) emissions and the Dutch Healthy Diet index 2015 for optimized diets of 1,747 Dutch adults aged 18–79 years with low, intermediate and high educational level, from the DNFCS 2019–2021. The horizontal and vertical axis show the absolute GHG emissions and DHD15 index. The numbers at each point represent the percent difference in diet costs between current and optimized diets. Filled diamonds represent the GHG emissions and DHD15 index of current diets. Filled triangles represent optimization with educational groups as peers. The lines between symbols are meant to recognize the pattern, they are not linear interpolations. *Filled rounds represent results of the secondary analysis using the total population as peers, showing larger reductions in GHG emissions and larger improvements in dietary quality (DHD15). For all models and educational subgroups, the most healthy diet has the highest GHG emissions and highest diet costs. LEL, low educational level; IEL, intermediate educational level; HEL, high educational level. Supplemental file S6 contains descriptive data related to the secondary analysis in which models were not stratified for educational subgroups.

A secondary analyses addressed how the stratification for educational peer subgroups influenced the results. Using the total population as peers, instead of using educational subgroups as peers, increased the options for making linear combinations of diets. The results consistently showed more pronounced reductions in GHG emissions and increases in DHD15 index for diets of all educational subgroups, while the diet costs were still similar compared to current diets (Figure 2). In the preferable optimized diets, DHD15 index were 121, 119, and 122, and GHG emissions were 3.48, 3.56, and 3.56 kg CO_2_-eq respectively for participants with LEL, IEL and HEL.

**Figure 3:**
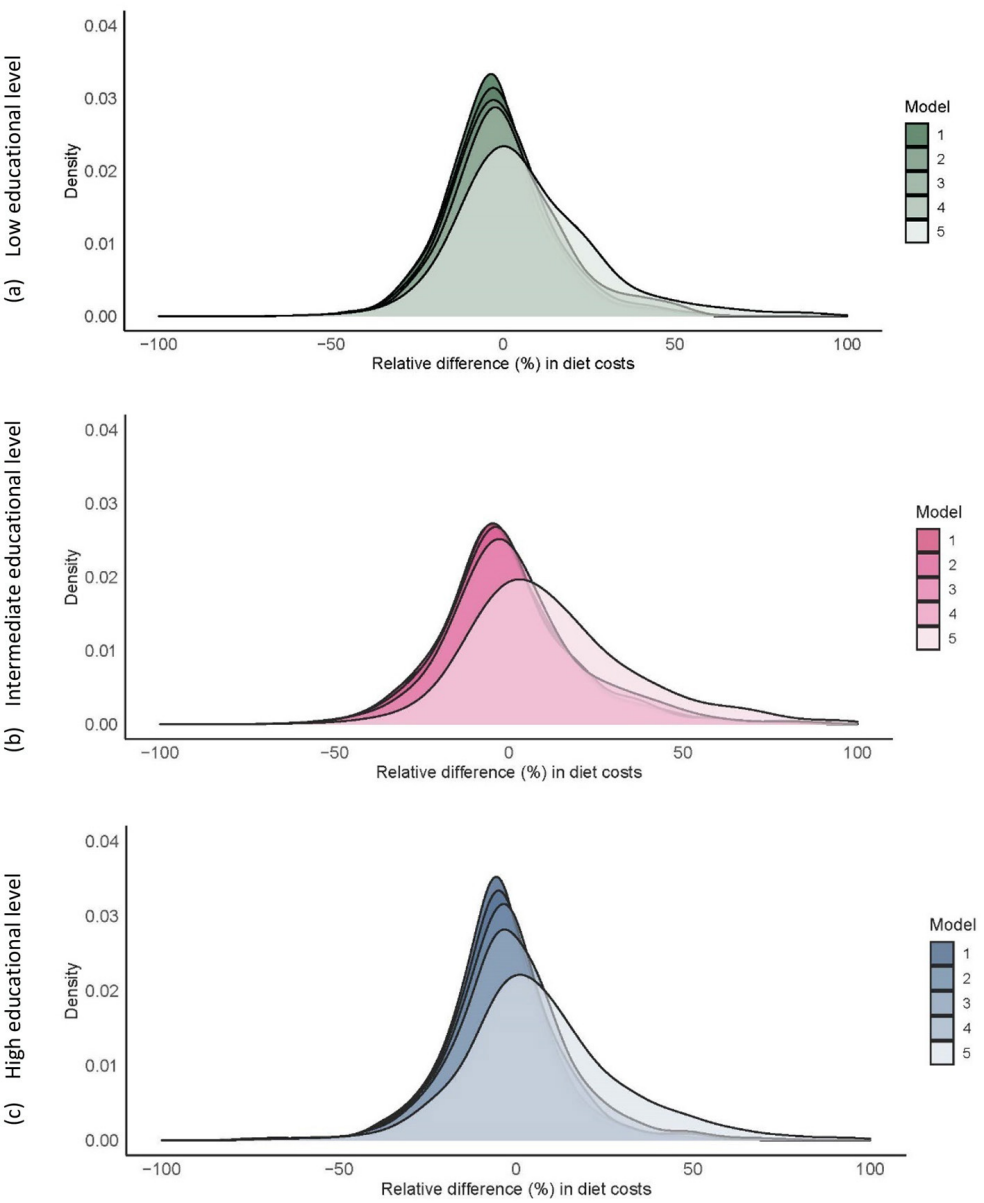
Relative (%) difference in diet costs between current and optimized diets (model 1–5) for 1,747 Dutch adults aged 18–79 years with low **(a)**, intermediate **(b)**, and high **(c)** educational level. Supplemental file S5 contains the distribution of the diet costs for the current and optimized diets.

The distribution of the relative (%) differences between costs of current and the optimized diets were approximately similar for all educational subgroups (Figures 3a–c).

### Diet composition of selected diets

**Figure 4:**
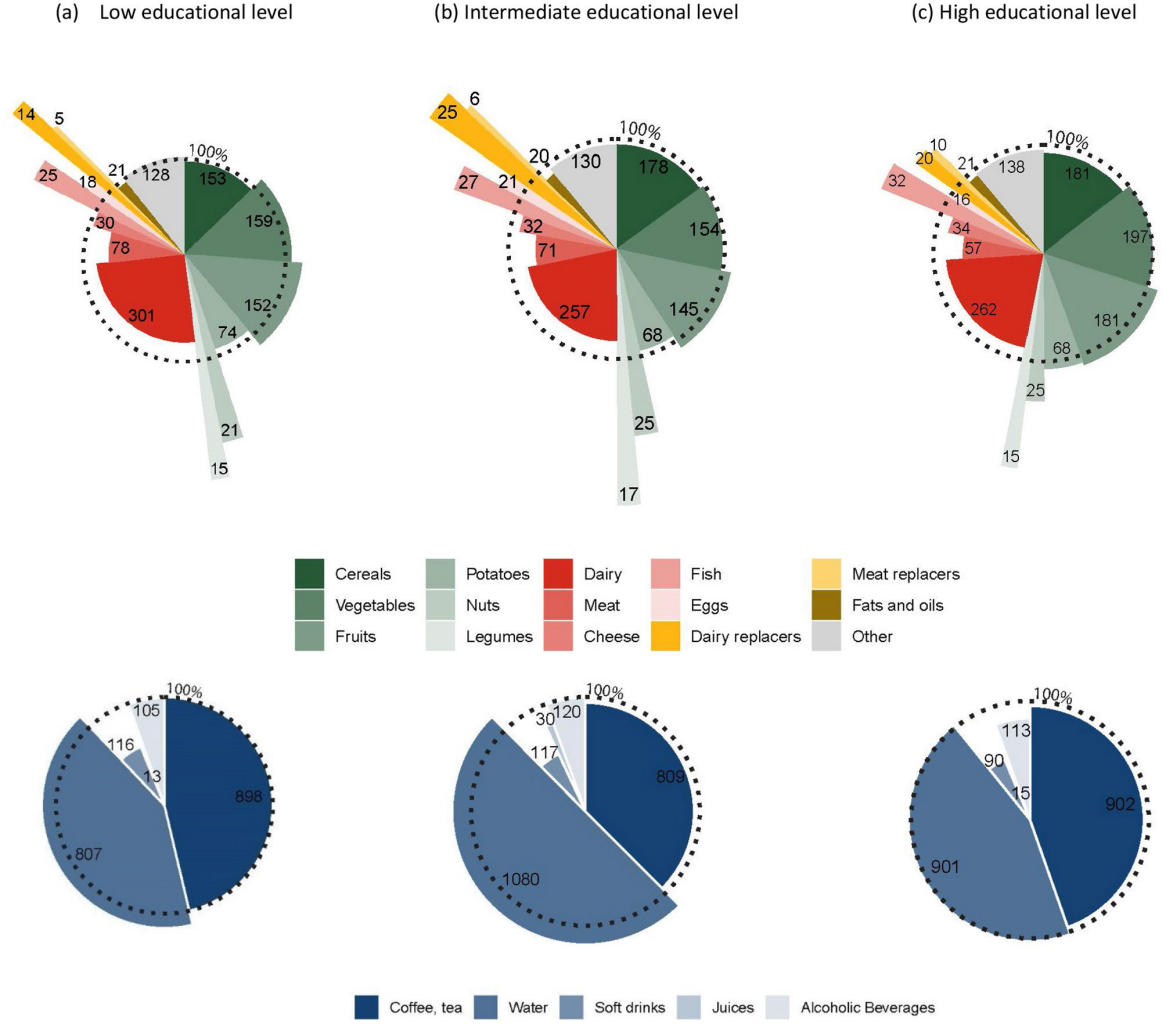
Share of foods (top) and beverages (bottom) in daily diets for the preferable optimized diets for 1,747 Dutch adults aged 18–79 years. The size of the sectors represent the amount of food in the preferable diets, whereas the radius represent the magnitude of relative difference between current and preferable diets for low **(a)**, intermediate **(b)**, and high **(c)** educational subgroups. The dotted round circle represent the current diet. Sectors outside the dotted circle indicate increased amounts, whereas sectors inside the dotted circle represents decreased amounts compared to current diets. Values presented in the sectors display the absolute amounts of foods (in g) in the diets. Supplemental file S3 contains descriptive data on food consumption in grams per day and relative differences for the current diet compared to optimized diets.

The preferable optimized diets for participants with LEL contained compared to their current diets, more fruits (increase of 16%, in total 152 g/day), vegetables (6%, 159 g), nuts (89%, 21 g, especially unsalted nuts), legumes (89%, 15 g), fish (66%, 25 g, with a shift from non-fatty to fatty fish), and eggs (13%, 18 g; Figure 4). Conversely, their diets contained less grains (−8%, 153 g) while shifting from refined to whole grains, dairy (−13%, 301 g), and meat (−25%, 78 g). The diets contained more water (13%; 807 g) and less soft drinks (−42%; 116 g) and juices (−51%; 13 g).

For participants with IEL, the preferable optimized diets contained compared to their current diets, more fruits (8%; 145 g), nuts (70%; 25 g, especially unsalted nuts), legumes (136%; 17 g), fish (60%; 27 g, especially fatty fish), and eggs (19%; 21 g). This was accompanied by lower amounts of grains (−5%; 178 g), potatoes (−5%; 68 g), dairy (−17%; 257 g), and meat (−25%; 71 g), and less cheese (−9%; 32 g). The diets contained more water (16%; 1,080 g) and less soft drinks (−45%; 117 g) and juices (−18%; 30 g).

The preferable optimized diets for participant with HEL contained compared to their current diets, more fruits (10%; 181 g), potatoes (7%; 68 g), nuts (37%; 25 g, especially unsalted nuts), legumes (100%; 15 g), and fish (59%; 32 g, mainly fatty fish). However, the diet contained lower amounts of grains (−8%; 181 g, with a shift from refined to whole grain), dairy (−12%; 262 g), meat (−27%; 57 g) and cheese (−11%; 34 g). Diets contained less soft drinks (−45%; 90 g), juices (−56%; 15 g) and alcoholic beverages (−15%; 113 g).

### Secondary environmental and nutritional outcomes of selected diets

The environmental indicators land use, acidification, and eutrophication of both freshwater and marine water, were lower in the preferable optimized diets compared to current diets for all educational subgroups (Supplemental file S2). In contrast, blue water usage of the preferable optimized diets increased by 15%, 7%, and 12%, respectively for the LEL, IEL, and HEL subgroups. Protein intake shifted toward more plant protein, with a most pronounced proportion of 45% for participants with HEL. Protein intake was set as a constraint at ±5% the total protein intake, and decreased by 3%−4% (Supplemental file S2). Across all educational subgroups, the preferable optimized diets contained less energy, (saturated) fat, mono and disaccharides, and sodium, and more EPA, DHA, and dietary fiber. Modest reductions of approximately 5% in micronutrients were observed (Supplemental file S4).

### Sensitivity analysis

Sensitivity analyses addressed the use of aggregated median and mean food prices instead of the minimal food prices, based on the lowest prices of barcodes. Using mean and median food prices, instead of the minimal food prices to calculate daily diet costs, did not alter our conclusions (Supplemental file S6).

## Discussion

Universal access to healthy, safe, and environmentally sustainable diets is essential across all socio-economic backgrounds to improve human and planetary health. Our results reveal that the costs for the preferable optimized diets were similar to the costs of current diets across all educational socio-economic subgroups, while being significantly more environmentally sustainable and healthier. Diets across low, intermediate, and high educational subgroups showed reductions in GHG emissions (respectively, 20%, 24%, and 19%) and improvements in diet quality according to the Dutch Healthy Diet 2015 (DHD15) index (respectively, 56%, 52%, and 53%). The preferable optimized diets of lower educational subgroups contained more vegetables (6%), while diets across all educational subgroups contained more fruit (10%−16%) and fewer grains (−5% to −8%), with a shift toward whole grains. Moreover, all diets contained approximately 50 g less dairy (−12% to −17%) and around 20–25 g less red and processed meat (−25% to −27%), and more unsalted nuts, legumes, and eggs as alternative protein sources. In the preferable optimized diets of low and intermediate educational subgroups, soft drinks and juices were partially substituted with water, while diets of high educational subgroups contained less alcoholic beverages. Although diets substantially improved, disparities in diet quality remained, with lower educational subgroups' diets containing fewer vegetables and fruits and more meat compared to higher subgroups. Greater improvements in environmental sustainability and diet quality are possible for low and intermediate educational subgroups, still without affecting diet costs, if the educational subgroups would be willing and able to adopt diets from outside their educational subgroup.

This study reveals that it is possible to achieve healthier and more sustainable diets without increasing diet costs across socio-economic subgroups. The diet optimization shows trade-offs as prioritizing diet quality resulted in less pronounced reductions in GHG emissions and higher diet costs while prioritizing GHG reductions lowered diet costs but had less pronounced improvements in diet quality. These findings align with a French study, which did not specifically analyse socio-economic positions (36). In addition, a Dutch modeling study found that healthy diets were associated with higher costs compared to current diets, which is consistent with our findings. However, sustainability considerations were not included in their analysis (37). Furthermore, previous studies found that when diets were optimized for nutritional adequacy or quality, the cost of those diets tended to increase, and GHG emissions did not consistently decrease (22, 23). Conversely, when diets were optimized for environmental sustainability or in case diet cost constraints were added, the quality of the diet tended to decline (22, 23, 38, 39). The application of varying models, objectives and constrains may explain these differences. However, the trade-offs underscore the need for an integrated approach taking into account health, environmental sustainability, and costs, to avoid unintended consequences of dietary changes while striving for sustainable diets.

The simultaneous improvement in environmental sustainability and diet quality, while maintaining diet costs, can be attributed to a shift in diet composition, primarily replacing meat with more plant-based foods. While these findings align with previous findings (36, 40), it should be noted that using the minimum food prices, reflecting the least costs principle, may underestimate real-world costs. In general, meat and fish, and fruits and vegetables are the most expensive dietary components due to their contribution to daily diets and elevated costs (14, 15, 41–43). In this study, the similar costs between the current and preferable optimized diets can be rationalized by reallocating expenses from meat to increased quantities of fruits and vegetables. This resulted in a net-zero cost effect across socio-economic subgroups.

Despite substantial improvements in the preferable optimized diets, disparities in diet quality persisted across educational subgroups, which is commonly observed between different levels of SEP (18, 19). Individuals with a lower educational level typically consume more processed meats, refined grains, and energy-dense foods and drinks (44), while those with a higher educational level select more vegetables, fruits, meat, and fish (45). In this study, the preferable optimized diets for lower educated subgroups included fewer vegetables (159 vs. 197 g) and more red and processed meat (78 vs. 57 g) compared to diets of higher educated subgroups, reflected into lower DHD15 index (109 vs. 118). Nonetheless, secondary analyses using the total population as peers, rather than subgroup peers, nearly eliminated disparities in diet quality between the educational subgroups, with respectively, 121 and 122 DHD15 index points for low and high educated subgroups. The use of broader peer groups increased the options of the model for making linear combinations, resulting in higher quality diets for lower and intermediate educational subgroups, primarily due to higher amounts of vegetables and fruits and lower amounts of meat. Though these more pronounced dietary shifts may be less acceptable or feasible for individuals with lower educational levels.

Previous research shows that differences in diet quality across SEP groups are largely driven by food costs (18, 19, 46), with low-income households having limited access to healthier foods (18, 19). While diet affordability is preferably assessed by the percentage of income spent on food, income data are unavailable in the DNFCS. However, Dutch income statistics show that low-income households spend 19% of their disposable income on food (€10,-/day) compared to 14% for the highest-income households (€23,-/day) (47, 48). This highlights the role of disparities in diet quality. Beyond economics, food accessibility and socio-cultural factors influence diet quality across SEP (18, 19). Individuals with lower SEP often may face unhealthier food environments and limited access to healthier options (18, 19), while cultural traditions and social support both may hinder and support healthier diets (18, 49).

Diet optimization models are valuable for understanding the socio-cultural, economic, and environmental synergies and trade-offs in the shift to sustainable diets. However, their validity relies on well-defined objectives and constraints (22, 23). In this study, diet acceptability was examined by taking the percentage deviations from current diets. While there is no agreed definition on how acceptability should be determined, deviation from current diets is assumed to be the most appropriate way to model acceptable diets (22). In addition, because no acceptability constraints were set for generally under-consumed but recommended food groups such as fatty fish (i.e., when baseline intake is often zero), our model led to a substantial increase in fish consumption (mainly higher fatty fish, lower lean fish). While this may benefit health, it does not consider overfishing or the sustainability of marine ecosystems, nor is this reflected in the LCA data. Future analyses should integrate constraints on fish consumption and account for overfishing, aiming to protect fish stocks. Moreover, while models may simplify real-world situations, studying SEP subgroups helps incorporate factors such as food preferences, and economical, behavioral or psychological influences on food choices (18, 19). Furthermore, educational level was used as a proxy for SEP, a multidimensional construct encompassing occupation, education and income (29). This can cause misclassification, as individuals with lower levels of education may hold high-earning professions, whereas individuals with higher levels of education may still be pursuing their studies and possess lower incomes. Nevertheless, convincing economic principles (50) and previous studies consistently link SEP measures to diet quality, even though the cross-sectional nature of nutritional studies limits causal inferences (18, 19). Further longitudinal and comprehensive research is needed to deepen the understanding of the complex interplay between socioeconomic factors and dietary patterns, while also taking into account disposal income, expenditures and affordability.

Ensuring equitable access to healthy, sustainable diets for all is a pivotal governmental responsibility in Western countries. While this study demonstrates the feasibility of the shift to healthy, sustainable and affordable diets, achieving this goal necessitates a facilitating food environment. In line with our study, research consistently shows that healthier foods are more expensive compared to less healthy foods, posing barriers to improved food choices (14, 15). Policy interventions, such as (dis)incentives, can improve access to health, sustainable diets (18), particularly when interventions are tailored to address disparities across SEP subgroups. For instance, reducing alcohol consumption among individuals with higher SEP and increasing vegetable consumption among individuals with lower SEP may require subgroup specific targeted strategies. In addition, tailored policies can support individuals with lower SEP to better understand health campaigns and dietary recommendations (18). Although significant dietary improvements are possible resulting in healthier, sustainable diets without additional costs, monitoring food prices is essential as rising costs may hinder the adoption of healthier diets, particularly for individuals with lower SEP. Lastly, despite the substantial improvements in diets, the optimized diets felt short of the Dutch government's 50/50 plant-to-animal protein target. The diets achieved respectively, 40%, 43%, and 45% plant-based protein in the preferable optimized diets for low, intermediate, and high educational subgroups (9). Achieving the policy target would require larger dietary changes, which may be less acceptable to individuals.

To conclude, our diet optimization modeling study demonstrates that, without increasing diet costs across all educational levels, both the environmental sustainability and diet quality of Dutch dietary patterns can be significantly improved. Environmental impact was reduced by 19%−24% and adherence to dietary guidelines increased by 52%−56%. Moreover, our analysis supports that factors associated with socio-economic position do contribute to the disparities in dietary quality.

## Data Availability

The data that support the findings of this study are available from the corresponding author upon reasonable request.
