# Supplementary material for: Diets optimized for environmental sustainability and health: implications for diet costs across socio-economic positions for Dutch adults
Source: Front Nutr. 2025 Nov 17;12:1667399. doi: 10.3389/fnut.2025.1667399 (PMC12665553; doi:10.3389/fnut.2025.1667399)
Supplement: Supplementary file 1 [file Supplementary_file_1.docx]

# SUPPLEMENTAL FILES

**Supplemental file 1**. SHARP model

**Supplemental file 2**. Summary output for optimized diets

**Supplemental file 3**. Food consumption and differences between current and optimized diets

**Supplemental file 4**. Macro and micronutrients for current and optimized diets

**Supplemental file 5**. Distribution of diet costs for current and optimized diets

**Supplemental file 6**. Outcomes diet optimization for secondary analysis, using the total population as peers

## Supplemental file 1 SHARP model

**Supplemental file 1.1 – Explanation SHARP model**

The objective functions, the main constraints, and the constraints used to calculate the diet quality index (Dutch Healthy Diet index 2015 (DHD15)) are presented and explained below. This model is used iteratively to calculate an optimized diet for each of the individuals in the sample. From now on the evaluated diet is indicated with an index *j’*.

**Objective functions**

Equation *(1)* is an objective function of the model, which minimizes the total GHG emissions of the optimized diet, expressed as a linear combination of GHG emissions of current diets.

| $min\left\{ F_{ghge}=\sum_{j} ghge_{j}{\cdot L}_{j} \right\}$ |  | (1) |
| --- | --- | --- |

Where $F_{ghge}$ is the total greenhouse gas (GHG) emissions of the optimized diet, $ghge_{j}$ is the GHG emissions of diet *j*, and $L_{j}$is the share of diet *j* in the optimized diet.

Objective function *(2)* maximizes the Dutch Healthy Diet index 2015 (DHD15-index) which is selected to be the health index in this study [47].

| $max\left\{ F_{heal}=\sum_{c} S_{c} \right\}$ |  | (2) |
| --- | --- | --- |

Where $F_{heal}$is the health quality index of the optimized diet, and $S_{c}$ is the partial health index score of food component *c*.

**Main constraints**

Constraints *(3)* are used to calculate the absolute deviation between food group consumption of the optimized and the current diet.

| $\sum_{j} q_{g,j}\cdot L_{j}{- D}_{g}^{+}+D_{g}^{-}=q_{g,j'}$ | $\forall g$ | (3) |
| --- | --- | --- |

Where $q_{g,j}$ is the consumption of food group *g* in diet *j*, $q_{g,j'}$ is the consumption level of food group *g* in the evaluated diet *j’*, $D_{g}^{+}$ is the positive deviation between the food group consumption of the optimized diet (i.e. $\sum_{j} q_{g,j}\cdot L_{j}$) and the current diet ($q_{g,j'}$), $D_{g}^{-}$ is the negative deviation between the food group consumption of the optimized diet and the current diet.

Equations *(4)* impose that for each food group the sum of the deviations is smaller than 33% of an individual’s current consumption, for all food groups *g* where consumption was more than 0 gram, ensuring that the optimized diet remains within realistic ranges.

| $D_{g}^{+}+D_{g}^{-}\leq0.33* \sum_{j} q_{g,j'}$ | $\forall g$ | (4) |
| --- | --- | --- |

Equations *(4a)* and *(4b)* impose that energy (kcal) and protein intake were set to be withing 5% of the observed intake.

| $\sum_{j} {ncnt}_{n,j}\cdot L_{j}\leq1.05*{ncnt}_{n,j'}$ | $\forall n$\|$n\in\{kcal,protein\}$ | (4a) |
| --- | --- | --- |
| $\sum_{j} {ncnt}_{n,j}\cdot L_{j}\geq0.95*{ncnt}_{n,j'}$ | $\forall n$\|$n\in\{kcal,protein\}$ | (4b) |

Where ${ncnt}_{n,j}$ is the content of nutrient *n* in diet *j*, ${ncnt}_{n,j'}$ is the content of nutrient *n* in the evaluated diet *j’*.

Equation *(5)* is the add up constraint imposing that the sum of the shares of current diets *j* in the optimized diet does not exceed 1.

| $\sum_{j} L_{j}=1$ |  | (5) |
| --- | --- | --- |

Constraints *(6)* impose that the partial health index scores of each food component in the optimized diet are larger or equal to the partial health index scores of the same food component in the current diet, i.e. ensuring the diet will be at least as healthy as the current diet in each food component.

| $S_{c}\geq s_{c}$ | $\forall c$ | (6) |
| --- | --- | --- |

Where $S_{c}$ is the food component score that corresponds to the optimized food component intake, $s_{c}$ is the food component score that corresponds to the current food component intake.

**Modelling the partial health scores**

The DHD15 index exists of 15 food components. There are four types of calculating the score for a food component in the DHD15 index, which are explained below.

Type 1: food component with a recommended minimum intake

The score of the components of the DHD15 index like the one presented in Supplemental figure 1 are modelled using constraints *(7)* and *(8)*. The components vegetables, fruit, whole grain products, legumes, nuts, fish (including max 4g lean fish), and tea are of this type. Constraint *(7)* imposes that the score increases from the minimum possible score (0) with a slope of *φ*. Constraint *(8)* imposes that the score of the component *c* is restricted to the maximum score of the specific component.

*b*

*maxS*

*Intake*

*Score (S)*

*a*

*φ*

Supplemental figure 1. Scoring function for food components of the Dutch Healthy Diet index 2015 that have a recommended minimum intake level.

| $S_{c}\leq\varphi_{c} *intake_{c}$ |  | (7) |
| --- | --- | --- |
| $S_{c}\leq maxS_{c}$ |  | (8) |

Where *a_c_* is the maximum food component intake level with the minimum individual health score, *b_c_* is the smallest intake level that receive an individual health score of 10, *φ*_c_ is the rate of change of the score between food component intake levels *a_c_* and *b_c_*, $maxS_{c}$ is the maximum possible individual score of the component (i.e. either 5 or 10), $intake_{c}$ is the intake in gram of the component *c* in the optimized diet.

Type 2: food components with a recommended maximum intake

The score of the components of the DHD15 index like the one presented in Supplemental figure 2 are modelled using constraints *(9)*-*(12)*. The components red meat, processed meat, sweetened beverages and fruit juices, alcohol, and sodium are of this type.

*b*

*maxS*

*Intake*

*Score (S)*

*a*

*φ*

*b*

*maxS*

*Intake*

*Score (S)*

*a*

*φ*

Supplemental figure 2. Scoring function for food components of the Dutch Healthy Diet index 2015 that have a recommended maximum intake level. The recommended maximum intake level can be either 0 (left) or some positive amount a (right).

| $Intake_{c}-b_{c}\leq bigM\cdot B_{c}$ |  | (9) |
| --- | --- | --- |
| $S_{c}\leq maxS_{c}- \varphi_{c}\left( intake_{c}-a_{c} \right)+ bigN\cdot B_{c}$ |  | (10) |
| $b_{c}-intake_{c}\leq b_{c}\cdot\left( 1-B_{c} \right)$ |  | (11) |
| $S_{c}\leq maxS_{c}\cdot\left( 1-B_{c} \right)$ |  | (12) |

Where *a_c_* is the maximum food component intake level with the maximum individual health score, *b_c_* is the smallest intake level that receive the minimum individual health score, *B_c_* is a binary variable that takes the value of 1 if the food component intake becomes larger than *b_c_* (and 0 otherwise), and *bigM* and *bigN* are very large numbers.

If the binary variable *B_c_* becomes 1 then constraints *(9)* and *(10)* become not binding while constraints *(11)* and *(12)* become binding and impose that the intake is greater than *b_c_* and the individual health score is set to 0. On the contrary if *B_c_* becomes 0 then constraints *(9)* and *(10)* become binding. Constraint *(9)* imposes that the food component intake is lower than *b_c_* and constraint *(10)* imposes that the score decreases from the maximum possible individual score with a slope *φ_c_*. For food component levels lower than a, the right hand side of constraint *(10)* become more than the maximum possible individual food component score. However because of constraint *(12)* the score value is restricted to the maximum possible score of the specific component.

Type 3: food components with a recommended range

The score of the components of the DHD15 index like the one presented in Supplemental figure 3 are modelled using constraints *(13)*-*(17)*. The component dairy (including max 40g cheese) is of this type.

*maxSc*

*Score (S)*

*Intake*

*θ*

*a*

*b*

$$\text{φ}$$

*c*

*d*

Supplemental figure 3. Scoring function for food components of the Dutch Healthy Diet index 2015 that have an optimum intake level.

| $Intake_{c}-d_{c}\leq bigM\cdot B_{c}$ |  | (13) |
| --- | --- | --- |
| $S_{c}\leq maxS_{c}- \theta_{c}\left( intake-c_{c} \right)+ bigN\cdot B_{c}$ |  | (14) |
| $d_{c}-intake\leq d_{c}\cdot\left( 1-B_{c} \right)$ |  | (15) |
| $S_{c}\leq maxS_{c}\cdot\left( 1-B_{c} \right)$ |  | (16) |
| $S_{c}\leq\varphi_{c} *intake_{c}$ |  | (17) |

Where *c_c_* is the maximum food component intake level with the maximum individual health score, and *d_c_* is the minimum food component intake level with the minimum individual health score.

If the binary variable *B_c_* becomes 1 then constraints *(13)* and *(14)* become not binding while constraints *(15)* and *(16)* become binding and impose that the intake is greater than *d_c_* and the individual health score is set to 0. On the contrary if *B_c_* becomes 0 then constraints *(13)* and *(14)* become binding. Constraint *(13)* imposes that the food component intake is lower than *d_c_* and constraint *(14)* imposes that the score decreases from the maximum possible individual score with a slope $\theta$. For food component levels lower than *c_c_* the right hand side of constraint *(14)* become more than the maximum possible individual food component score. However because of constraint *(16)* the score value is restricted to the maximum possible score of the specific component *c*. Constraint *(17)* imposes that the score increases from the minimum possible score (0) with a slope of $\varphi_{c}$, again restricted by constraint *(16)* to the maximum possible score of the specific component.

Type 4: food components with a recommended replacement (ratio)

The DHD15 score comprises of ratio components like the ‘Replace refined with wholegrain products’ and ‘Replace butter and hard fats with margarines and oils’ component. The score of such components are presented in Supplemental figure 4 and are modelled using constraints *(18)*-*(20)*. To approximate ratio components in a mixed integer linear programming model we assumed that the individual score function of a ratio component remain the same between specific intake levels *(m)*. By increasing the number of intake levels we achieved a rather accurate approximation of the score function of such components.

*Score (S)*

$$e_{3}$$

*XR/YR*

$$e_{1}$$

$$e_{m}$$

${Score}_{c,4}$

*maxS*

Supplemental figure 4. Scoring function for food components of the Dutch Healthy Diet index 2015 that include a ratio.

| $e_{c,m}*YR_{c}-XR_{c}\leq bigM *(1-Y_{c,m})$ | $\forall m$ | (18) |
| --- | --- | --- |
| $\sum_{m} Y_{c,m}=1$ |  | (19) |
| $S_{c}=\sum_{m} {Score}_{c,m} *Y_{c,m}$ |  | (20) |

Where XR is the intake of the nutrient or food group that is on the numerator of the ratio component (e.g. the intake of whole grains in the optimized diet), YR is the intake of the nutrient or food group that is on the denominator (e.g. the intake of refined grains in the optimized diet), $e_{m}$ is the level of XR/YR that receives the *score_c,m_*, $Y_{c,m}$ is a binary variable that takes the value of 1 if the value of the ratio component XR/YR is larger than $e_{m}$ (and 0 otherwise).

Equation *(18)* imposes that the binary variable $Y_{c,m}$ becomes 1 if $e_{m}$ is smaller than XR/YR. Equation *(19)* ensures that only one score level can be selected. Equation *(20)* calculates the score of ratio components of the DHD15 index.

The components fish and dairy have some additional constraints which are reflected in equations *(21-25)*.

| $intake_{sc}-maxL_{sc}\leq{BV}_{sc}*{maxC}_{sc}$ | $\forall c \vert c \in\{fish,dairy\}$ | (21) |
| --- | --- | --- |
| $intake_{c}\leq intake_{c'}+maxL_{sc}+(1-{BV}_{sc})*{maxC}_{sc}$ | $\forall c \vert c \in\{fish,dairy\}$ | (22) |
| $intake_{c}\geq intake_{c'}+maxL_{sc}-(1-{BV}_{sc})*{maxC}_{sc}$ | $\forall c \vert c \in\{fish,dairy\}$ | (23) |
| $intake_{c}\leq intake_{c'}+{intake}_{sc}+{BV}_{sc}*{maxC}_{sc}$ | $\forall c \vert c \in\{fish,dairy\}$ | (24) |
| $intake_{c}\geq intake_{c'}+intake_{sc}-{BV}_{sc}*{maxC}_{sc}$ | $\forall c \vert c \in\{fish,dairy\}$ | (25) |

Where $intake_{sc}$ is the intake in gram of sub food group *s* within food component *c* in the optimized diet. $maxL_{sc}$ is the maximum level of sub food group *s* within component *c*. For example, within the component fish, a maximum level of 4g of lean fish is allowed. ${BV}_{c}$ is a binary variable that takes the value of 1 if $intake_{sc}$ becomes larger than $maxL_{sc}$ (constraint *(21)*). ${maxC}_{sc}$ is the maximum observed consumed amount in the dataset of sub food group *s* within the component *c*. $intake_{c'}$ is the intake of component *c* without the intake of sub food group *s* in the optimized diet.

If the binary variable *BV_sc_* becomes 1 then constraints *(22)* and *(23)* become binding and impose that the sub food group *s* intake is greater than $maxL_{sc}$ and the intake of the sub food group is set to this $maxL_{sc}$, while constraints *(24)* and *(25)* become not binding. On the contrary if *BV_c_* becomes 0 then constraints *(24)* and *(25)* become binding and impose that the sub food group intake is equal to the original intake.

Finally, constraints *(26)* are the domain specific constraints of the decision variables.

| ${intake_{c} \geq0 \forall c, intake_{sc}\geq0 \forall s,c, L}_{j}\geq0 \forall j, D_{g}^{+}\geq0 \forall g, D_{g}^{-}\geq0 \forall g, S_{c}\geq0 \forall c , B_{c} \in\left( 0,1 \right) \forall c, Y_{c,m}\in\left( 0,1 \right) \forall c,m, {BV}_{sc} \in\left( 0,1 \right) \forall c \vert c \in\{fish,dairy\}$ |  | (26) |
| --- | --- | --- |

**References**

1. Looman M, Feskens EJ, de Rijk M, Meijboom S, et al. (2017). Development and evaluation of the Dutch Healthy Diet index 2015. *Public Health Nutrition* **20**(13): 2289-2299. DOI: 10.1017/S136898001700091X.


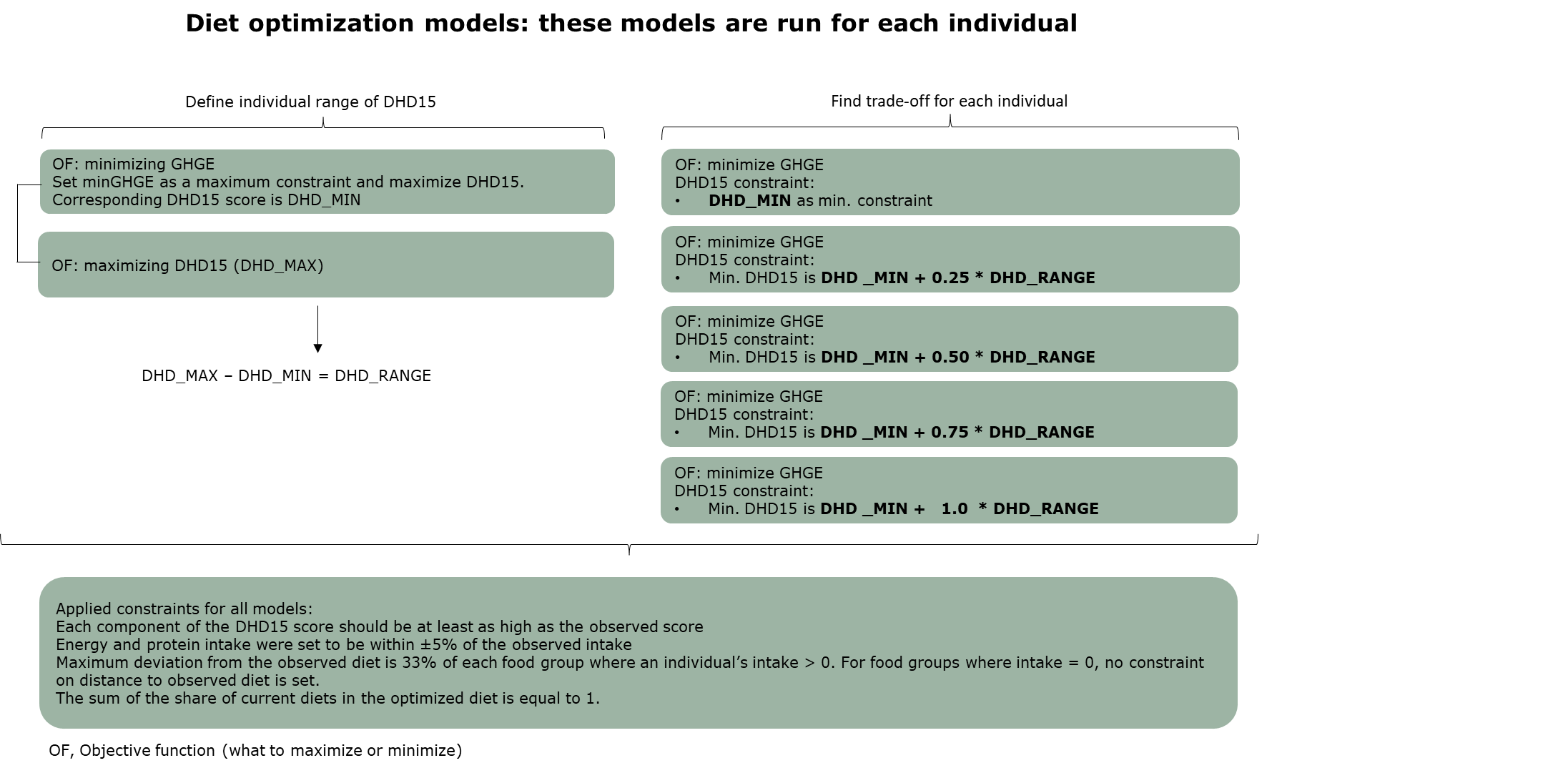


**Supplemental figure 1.1.** Steps of diet optimization models.

## Supplemental file 2. Summary output for optimized diets

**Table 2.1** Summary output for characteristics of optimized diets for 445 Dutch adults 18-79 years with low educational level, from the Dutch National Food Consumption Survey 2019-2021.

|  | **Low educational level** | | | | | | | | | | | | | |  |
| --- | --- | --- | --- | --- | --- | --- | --- | --- | --- | --- | --- | --- | --- | --- | --- |
|  | **Model 1** | | | **Model 2** | | | **Model 3** | | | **Model 4** | | | **Model 5** | | |
| Diet costs (€/day) based on |  |  |  |  |  |  |  |  |  |  |  |  |  |  |  |
| Minimum price | 3.02 | (2.43- | 3.72) | 3.04 | (2.46- | 3.72) | 3.10 | (2.50- | 3.77) | 3.14 | (2.57- | 3.82) | 3,28 | (2,78- | 3,92) |
| Mean price | 7.05 | (5.80- | 8.64) | 7.13 | (5.89- | 8.67) | 7.21 | (5.99- | 8.76) | 7.32 | (6.13- | 8.78) | 7,74 | (6,59- | 9,05) |
| Median price | 6.76 | (5.57- | 8.24) | 6.85 | (5.66- | 8.26) | 6.90 | (5.75- | 8.35) | 7.02 | (5.89- | 8.41) | 7,38 | (6,29- | 8,67) |
|  |  |  |  |  |  |  |  |  |  |  |  |  |  |  |  |
|  |  |  |  |  |  |  |  |  |  |  |  |  |  |  |  |
| DHD15 (points) | 92 | (79- | 103) | 98 | (84- | 109) | 104 | (90- | 115) | 109 | (96- | 121) | 116 | (101- | 128) |
| Energy (kcal) | 1900 | (619) |  | 1901 | (618) |  | 1904 | (616) |  | 1910 | (614) |  | 1932 | (609) |  |
| Protein (g) | 76 | (27) |  | 77 | (27) |  | 77 | (27) |  | 78 | (26) |  | 79 | (26) |  |
|  |  |  |  |  |  |  |  |  |  |  |  |  |  |  |  |
| Plant protein (%) | 41% |  |  | 40% |  |  | 40% |  |  | 40% |  |  | 38% |  |  |
|  |  |  |  |  |  |  |  |  |  |  |  |  |  |  |  |
| GHG emissions (kg CO_2_-eq/d) | 3.65 | (2.97- | 4.67) | 3.67 | (2.99- | 4.69) | 3.72 | (3.02- | 4.72) | 3.83 | (3.12- | 4.82) | 4,49 | (3,62- | 5,37) |
| Land use (m^2^*yr/d) | 2.42 | (2.00- | 3.04) | 2.44 | (2.01- | 3.05) | 2.46 | (2.02- | 3.05) | 2.52 | (2.08- | 3.10) | 2,73 | (2,24- | 3,32) |
| Freshwater eutrophication (g P-eq/d) | 0.30 | (0.25- | 0.38) | 0.30 | (0.25- | 0.37) | 0.30 | (0.25- | 0.38) | 0.31 | (0.26- | 0.38) | 0,33 | (0,28- | 0,40) |
| Marine water eutrophication (g N-eq/d) | 5.43 | (4.40- | 7.04) | 5.47 | (4.42- | 7.05) | 5.51 | (4.45- | 7.12) | 5.62 | (4.58- | 7.30) | 6,88 | (5,39- | 8,70) |
| Acidification (g SO_2_-eq/d) | 31.93 | (25.01- | 41.53) | 32.09 | (25.19- | 41.80) | 32.28 | (25.38- | 42.31) | 33.12 | (26.33- | 43.33) | 40,98 | (31,77- | 51,46) |
| Blue water (m^3^/d) | 0.13 | (0.10- | 0.18) | 0.14 | (0.10- | 0.18) | 0.14 | (0.11- | 0.19) | 0.15 | (0.12- | 0.19) | 0,17 | (0,13- | 0,20) |
|  |  |  |  |  |  |  |  |  |  |  |  |  |  |  |  |
| PREF | 673 | (450- | 927) | 688 | (483- | 932) | 717 | (501- | 951) | 743 | (546- | 982) | 815 | (609- | 1046) |
|  |  |  |  |  |  |  |  |  |  |  |  |  |  |  |  |

*Values are presented as proportions, means (SD) and medians (25^th-^75^th^ percentile).*

*DHD15, Dutch Healthy Diet 2015 index; PREF, preferences score based on deviation from current diets*

**Table 2.2.** Summary output for characteristics of optimized diets for 663 Dutch adults 18-79 years with intermediate educational level, from the Dutch National Food Consumption Survey 2019-2021.

|  | **Intermediate educational level** | | | | | | | | | | | | | |  |
| --- | --- | --- | --- | --- | --- | --- | --- | --- | --- | --- | --- | --- | --- | --- | --- |
|  | **Model 1** | | | **Model 2** | | | **Model 3** | | | **Model 4** | | | **Model 5** | | |
| Diet costs (€/day) based on |  |  |  |  |  |  |  |  |  |  |  |  |  |  |  |
| Minimum price | 3.18 | (2.66- | 3.73) | 3.23 | (2.70- | 3.75) | 3.24 | (2.69- | 3.79) | 3.30 | (2.79- | 3.87) | 3.62 | (3.08- | 4.32) |
| Mean price | 8.03 | (6.80- | 9.25) | 7.98 | (6.82- | 9.17) | 7.84 | (6.64- | 9.13) | 7.79 | (6.64- | 9.15) | 8.48 | (7.29- | 9.89) |
| Median price | 7.71 | (6.57- | 8.95) | 7.69 | (6.59- | 8.85) | 7.55 | (6.38- | 8.73) | 7.47 | (6.33- | 8.70) | 8.08 | (6.96- | 9.41) |
|  |  |  |  |  |  |  |  |  |  |  |  |  |  |  |  |
|  |  |  |  |  |  |  |  |  |  |  |  |  |  |  |  |
| DHD15 (points) | 81 | (71- | 91) | 91 | (81- | 99) | 101 | (90- | 109) | 111 | (100- | 120) | 121 | (108- | 131) |
| Energy (kcal) | 1983 | (675) |  | 1985 | (674) |  | 1984 | (673) |  | 1987 | (671) |  | 2019 | (660) |  |
| Protein (g) | 78 | (29) |  | 78 | (29) |  | 78 | (29) |  | 79 | (29) |  | 81 | (29) |  |
|  |  |  |  |  |  |  |  |  |  |  |  |  |  |  |  |
| Plant protein (%) | 45% |  |  | 45% |  |  | 44% |  |  | 43% |  |  | 40% |  |  |
|  |  |  |  |  |  |  |  |  |  |  |  |  |  |  |  |
| GHG emissions (kg CO_2_-eq/d) | 3.36 | (2.62- | 4.37) | 3.39 | (2.65- | 4.41) | 3.46 | (2.71- | 4.47) | 3.64 | (2.88- | 4.65) | 4.61 | (3.74- | 5.64) |
| Land use (m^2^*yr/d) | 2.30 | (1.78- | 2.89) | 2.32 | (1.82- | 2.91) | 2.37 | (1.86- | 2.94) | 2.48 | (1.96- | 3.05) | 2.81 | (2.27- | 3.42) |
| Freshwater eutrophication (g P-eq/d) | 0.29 | (0.22- | 0.37) | 0.29 | (0.23- | 0.37) | 0.30 | (0.23- | 0.37) | 0.31 | (0.25- | 0.39) | 0.35 | (0.29- | 0.43) |
| Marine water eutrophication (g N-eq/d) | 4.84 | (3.74- | 6.37) | 4.88 | (3.80- | 6.42) | 4.97 | (3.88- | 6.53) | 5.27 | (4.14- | 6.85) | 6.96 | (5.42- | 8.69) |
| Acidification (g SO_2_-eq/d) | 27.91 | (20.93- | 37.71) | 28.28 | (21.10- | 38.06) | 28.66 | (21.78- | 38.45) | 30.35 | (23.17- | 40.06) | 40.95 | (30.90- | 52.08) |
| Blue water (m^3^/d) | 0.13 | (0.10- | 0.17) | 0.14 | (0.10- | 0.18) | 0.15 | (0.11- | 0.18) | 0.16 | (0.13- | 0.20) | 0.19 | (0.16- | 0.23) |
|  |  |  |  |  |  |  |  |  |  |  |  |  |  |  |  |
| PREF | 921 | (713- | 1150) | 919 | (718- | 1146) | 912 | (702- | 1135) | 908 | (713- | 1144) | 940 | (740- | 1176) |
|  |  |  |  |  |  |  |  |  |  |  |  |  |  |  |  |

*Values are presented as proportions, means (SD) and medians (25^th-^75^th^ percentile).*

*DHD15, Dutch Healthy Diet 2015 index; PREF, preferences score based on deviation from current diets*

**Table 2.3.** Summary output for characteristics of optimized diets for 669 Dutch adults 18-79 years with high educational level, from Dutch National Food Consumption Survey 2019-2021.

|  | **High educational level** | | | | | | | | | | | | | |  |
| --- | --- | --- | --- | --- | --- | --- | --- | --- | --- | --- | --- | --- | --- | --- | --- |
|  | **Model 1** | | | **Model 2** | |  | **Model 3** | | | **Model 4** | | | **Model 5** | | |
| Diet costs (€/day) based on |  |  |  |  |  |  |  |  |  |  |  |  |  |  |  |
| Minimum price | 3.23 | (2.58 - | 4.02) | 3.26 | (2.61 - | 4.04) | 3.31 | (2.65 - | 4.05) | 3.36 | (2.73 - | 4.14) | 3.65 | (3.06 - | 4.43) |
| Mean price | 7.79 | (6.40 - | 9.56) | 7.83 | (6.51 - | 9.60) | 7.87 | (6.57 - | 9.63) | 7.98 | (6.66 - | 9.76) | 8.63 | (7.37 - | 10.35) |
| Median price | 7.39 | (6.09 - | 9.12) | 7.44 | (6.17 - | 9.14) | 7.48 | (6.25 - | 9.17) | 7.59 | (6.35 - | 9.32) | 8.19 | (7.02 - | 9.85) |
|  |  |  |  |  |  |  |  |  |  |  |  |  |  |  |  |
|  |  |  |  |  |  |  |  |  |  |  |  |  |  |  |  |
| DHD15 (points) | 97 | (85 - | 109) | 104 | (92 - | 114) | 111 | (98 - | 120) | 118 | (104 - | 126) | 125 | (110 - | 134) |
| Energy (kcal) | 2011 | (689) |  | 2013 | (686) |  | 2012 | (688) |  | 2020 | (691) |  | 2046 | (682) |  |
| Protein (g) | 77 | (28) |  | 77 | (27) |  | 77 | (27) |  | 78 | (28) |  | 80 | (27) |  |
|  |  |  |  |  |  |  |  |  |  |  |  |  |  |  |  |
| Plant protein (%) | 45% |  |  | 45% |  |  | 45% |  |  | 45% |  |  | 43% |  |  |
|  |  |  |  |  |  |  |  |  |  |  |  |  |  |  |  |
| GHG emissions (kg CO_2_-eq/d) | 3.63 | (2.93 - | 4.62) | 3.65 | (2.95 - | 4.66) | 3.68 | (2.98 - | 4.70) | 3.81 | (3.07 - | 4.82) | 4.53 | (3.65 - | 5.47) |
| Land use (m^2^*yr/d) | 2.41 | (1.96 - | 2.95) | 2.42 | (1.97 - | 2.97) | 2.45 | (2.00 - | 3.00) | 2.51 | (2.05 - | 3.06) | 2.77 | (2.28 - | 3.30) |
| Freshwater eutrophication (g P-eq/d) | 0.29 | (0.24 - | 0.36) | 0.29 | (0.24 - | 0.36) | 0.30 | (0.24 - | 0.37) | 0.30 | (0.25 - | 0.37) | 0.34 | (0.28 - | 0.41) |
| Marine water eutrophication (g N-eq/d) | 5.15 | (4.22 - | 6.57) | 5.16 | (4.22 - | 6.60) | 5.20 | (4.26 - | 6.64) | 5.30 | (4.36 - | 6.77) | 6.42 | (5.00 - | 8.08) |
| Acidification (g SO_2_-eq/d) | 29.60 | (23.12 - | 38.94) | 29.68 | (23.24 - | 39.03) | 30.02 | (23.57 - | 39.32) | 30.77 | (24.27 - | 40.22) | 37.47 | (28.43 - | 48.50) |
| Blue water (m^3^/d) | 0.17 | (0.13 - | 0.21) | 0.17 | (0.14 - | 0.21) | 0.18 | (0.14 - | 0.22) | 0.19 | (0.15 - | 0.22) | 0.21 | (0.18 - | 0.25) |
|  |  |  |  |  |  |  |  |  |  |  |  |  |  |  |  |
| PREF | 754 | (515 - | 991) | 762 | (544 - | 1007) | 796 | (563 - | 1025) | 826 | (590 - | 1062) | 885 | (646 - | 1148) |
|  |  |  |  |  |  |  |  |  |  |  |  |  |  |  |  |

*Values are presented as proportions, means (SD) and medians (25^th-^75^th^ percentile).*

*DHD15, Dutch Healthy Diet 2015 index; PREF, preferences score based on deviation from current diet*

## Supplemental file 3. Food consumption and differences between current and optimized diets.

**Supplemental table 3.1.** Daily food consumption in grams and relative (%) difference with current diets, for 445 Dutch adults 18-79 years with low educational level, from the Dutch National Food Consumption Survey 2019-2021.

|  | **Current** | **Model 1** | | **Model 2** | | **Model 3** | | **Model 4** | | **Model 5** | |
| --- | --- | --- | --- | --- | --- | --- | --- | --- | --- | --- | --- |
|  | Mean | Mean |  | Mean |  | Mean |  | Mean |  | Mean |  |
|  | (grams) | (grams) | Δ | (grams) | Δ | (grams) | Δ | (grams) | Δ | (grams) | Δ |
| **PLANT-BASED FOOD GROUPS** | |  |  |  |  |  |  |  |  |  |  |
| **Grains and grain-based products** | |  |  |  |  |  |  |  |  |  |  |
| Bread, rusks, wholegrain | 79 | 89 | 14% | 88 | 12% | 87 | 11% | 85 | 8% | 80 | 2% |
| Bread, rusks, refined | 35 | 25 | -28% | 24 | -31% | 23 | -33% | 22 | -37% | 21 | -39% |
| Flours, rice, grains, refined | 40 | 24 | -39% | 24 | -40% | 24 | -40% | 23 | -42% | 22 | -44% |
| Flours, rice, grains, wholegrain | 7 | 9 | 41% | 10 | 55% | 11 | 65% | 12 | 76% | 15 | 132% |
| Breakfast cereals, wholegrain | 5 | 7 | 39% | 8 | 45% | 8 | 51% | 8 | 63% | 8 | 60% |
| Breakfast cereals, refined | 2 | 2 | 9% | 2 | 20% | 2 | 23% | 2 | 22% | 2 | -3% |
| **Vegetables** | 150 | 146 | -3% | 149 | -1% | 153 | 2% | 159 | 6% | 169 | 13% |
| **Fruit** |  |  |  |  |  |  |  |  |  |  |  |
| Fruit, other | 50 | 53 | 8% | 55 | 12% | 58 | 17% | 60 | 22% | 64 | 29% |
| Apples and pears | 35 | 38 | 8% | 39 | 11% | 40 | 15% | 43 | 24% | 45 | 29% |
| Citrus fruits | 27 | 26 | -4% | 26 | -4% | 27 | -2% | 28 | 2% | 31 | 12% |
| Berries | 11 | 7 | -37% | 7 | -34% | 8 | -31% | 9 | -23% | 15 | 32% |
| Fruit compote | 5 | 5 | 0% | 5 | 5% | 6 | 10% | 6 | 18% | 7 | 37% |
| Mixed fruits and olives | 3 | 4 | 43% | 5 | 67% | 5 | 86% | 6 | 114% | 7 | 148% |
| **Potatoes and tubers** | 76 | 74 | -2% | 73 | -3% | 73 | -3% | 74 | -2% | 74 | -3% |
| **Nuts and seeds** |  |  |  |  |  |  |  |  |  |  |  |
| Nuts and seeds, not unsalted | 6 | 9 | 45% | 9 | 43% | 9 | 39% | 8 | 35% | 9 | 35% |
| Nuts and seeds, unsalted | 5 | 10 | 113% | 11 | 124% | 11 | 136% | 13 | 154% | 12 | 152% |
| **Legumes** | 7 | 9 | 31% | 11 | 71% | 14 | 108% | 15 | 127% | 16 | 137% |
|  |  |  |  |  |  |  |  |  |  |  |  |
| **ANIMAL-BASED FOOD GROUPS** |  |  |  |  |  |  |  |  |  |  |  |
| **Dairy** |  |  |  |  |  |  |  |  |  |  |  |
| Milk and yoghurt products, sugar free | 263 | 232 | -12% | 234 | -11% | 236 | -10% | 242 | -8% | 249 | -5% |
| Milk and yoghurt products, sugared | 16 | 8 | -48% | 8 | -50% | 8 | -51% | 7 | -52% | 7 | -55% |
| Dairy, other | 69 | 53 | -22% | 53 | -23% | 53 | -23% | 52 | -24% | 57 | -17% |
| **Meat** |  |  |  |  |  |  |  |  |  |  |  |
| Meat, hot meal, processed | 30 | 21 | -30% | 21 | -32% | 20 | -36% | 17 | -43% | 13 | -56% |
| Meat, cold meal, processed | 24 | 15 | -35% | 15 | -36% | 15 | -37% | 14 | -39% | 12 | -48% |
| Meat beef, unprocessed | 17 | 6 | -63% | 6 | -62% | 7 | -60% | 7 | -55% | 16 | -1% |
| Meat poultry, unprocessed | 16 | 18 | 11% | 18 | 13% | 19 | 17% | 20 | 24% | 22 | 35% |
| Meat pork, unprocessed | 14 | 16 | 12% | 16 | 10% | 16 | 11% | 16 | 15% | 14 | 1% |
| Meat, other | 3 | 1 | -42% | 1 | -43% | 1 | -43% | 2 | -38% | 2 | -9% |
| **Cheese** | 32 | 30 | -5% | 30 | -5% | 30 | -5% | 30 | -6% | 31 | -4% |
| **Fish** |  |  |  |  |  |  |  |  |  |  |  |
| Fish, not fatty | 9 | 8 | -12% | 8 | -12% | 8 | -12% | 8 | -11% | 8 | -12% |
| Fish, fatty | 6 | 12 | 110% | 14 | 139% | 15 | 168% | 17 | 190% | 17 | 193% |
| **Eggs** | 16 | 18 | 13% | 18 | 11% | 17 | 9% | 18 | 10% | 18 | 15% |
| **Dairy and meat replacers** |  |  |  |  |  |  |  |  |  |  |  |
| Dairy replacers | 6 | 12 | 91% | 12 | 91% | 13 | 98% | 14 | 117% | 14 | 121% |
| Meat replacers | 3 | 6 | 124% | 6 | 109% | 6 | 97% | 5 | 90% | 5 | 70% |
|  |  |  |  |  |  |  |  |  |  |  |  |
| **MIXED FOOD GROUPS** |  |  |  |  |  |  |  |  |  |  |  |
| **Soups and bouillon** | 22 | 21 | -6% | 22 | -2% | 23 | 2% | 23 | 5% | 24 | 7% |
| **Biscuits and sweet pastries** | 42 | 43 | 1% | 42 | 0% | 41 | -2% | 41 | -3% | 41 | -3% |
| **Contaminants, yeast and sauces** | 34 | 30 | -14% | 30 | -14% | 30 | -13% | 30 | -14% | 31 | -11% |
| **Sugar and confectionary** |  |  |  |  |  |  |  |  |  |  |  |
| Sugar, honey, jam, syrup, dessert sauce | 11 | 10 | -9% | 10 | -9% | 10 | -9% | 10 | -10% | 10 | -12% |
| Chocolate, candy bars, chocolate bread toppings | 8 | 9 | 14% | 9 | 12% | 9 | 11% | 9 | 10% | 8 | 7% |
| Sweets without chocolate, other bread toppings | 2 | 4 | 63% | 4 | 55% | 4 | 49% | 3 | 40% | 3 | 39% |
| **Fats and oils** |  |  |  |  |  |  |  |  |  |  |  |
| Fats and oils, soft | 19 | 18 | -3% | 18 | -3% | 18 | -3% | 18 | -3% | 18 | -6% |
| Fats and oils, hard | 4 | 3 | -33% | 3 | -33% | 3 | -32% | 3 | -33% | 3 | -34% |
| **Savoury snacks** |  |  |  |  |  |  |  |  |  |  |  |
| Pretzels, chips, salty biscuits | 6 | 6 | -3% | 6 | -5% | 6 | -5% | 5 | -11% | 5 | -17% |
| Snacks, croquettes, snack rolls | 6 | 7 | 1% | 6 | -1% | 6 | -4% | 6 | 1% | 8 | 22% |
|  |  |  |  |  |  |  |  |  |  |  |  |
| **BEVERAGES** |  |  |  |  |  |  |  |  |  |  |  |
| **Non-alcoholic beverages** |  |  |  |  |  |  |  |  |  |  |  |
| Coffee and tea | 898 | 848 | -6% | 852 | -5% | 865 | -4% | 898 | 0% | 920 | 2% |
| Water | 713 | 809 | 14% | 811 | 14% | 809 | 14% | 807 | 13% | 809 | 13% |
| Soft drinks, sugared | 121 | 69 | -43% | 67 | -45% | 64 | -47% | 61 | -49% | 58 | -52% |
| Soft drinks, sugar free | 61 | 33 | -47% | 33 | -47% | 33 | -46% | 35 | -42% | 48 | -21% |
| Alcohol free beverages | 17 | 20 | 19% | 20 | 18% | 20 | 18% | 20 | 14% | 17 | -4% |
| Juice, fruit | 25 | 13 | -49% | 12 | -49% | 12 | -50% | 12 | -51% | 12 | -50% |
| Juice, vegetables | 2 | 1 | -62% | 1 | -62% | 1 | -64% | 1 | -60% | 1 | -43% |
| **Alcoholic beverages** |  |  |  |  |  |  |  |  |  |  |  |
| Beer | 66 | 72 | 8% | 70 | 6% | 69 | 5% | 70 | 6% | 71 | 7% |
| Wine | 34 | 23 | -32% | 25 | -25% | 27 | -20% | 29 | -12% | 40 | 19% |
| Alcohol, other than beer wine | 6 | 5 | -24% | 5 | -21% | 5 | -19% | 5 | -19% | 5 | -15% |

**Supplemental table 3.2.** Daily food consumption in grams and relative (%) difference with current diets, for 663 Dutch adults 18-79 years with intermediate educational level, from the Dutch National Food Consumption Survey 2019-2021.

|  | **Current** | **Model 1** | | **Model 2** | | **Model 3** | | **Model 4** | | **Model 5** | |
| --- | --- | --- | --- | --- | --- | --- | --- | --- | --- | --- | --- |
|  | Mean | Mean |  | Mean |  | Mean |  | Mean |  | Mean |  |
|  | (grams) | (grams) | Δ | (grams) | Δ | (grams) | Δ | (grams) | Δ | (grams) | Δ |
| **PLANT-BASED FOOD GROUPS** |  |  |  |  |  |  |  |  |  |  |  |
| **Grains and grain-based products** |  |  |  |  |  |  |  |  |  |  |  |
| Bread, rusks, wholegrain | 83 | 79 | -5% | 80 | -4% | 81 | -3% | 80 | -3% | 74 | -11% |
| Bread, rusks, refined | 37 | 39 | 6% | 37 | 2% | 35 | -4% | 31 | -16% | 22 | -39% |
| Flours, rice, grains, refined | 50 | 43 | -15% | 41 | -18% | 41 | -19% | 42 | -17% | 39 | -23% |
| Flours, rice, grains, wholegrain | 8 | 9 | 11% | 9 | 14% | 10 | 22% | 11 | 39% | 21 | 151% |
| Breakfast cereals, wholegrain | 7 | 10 | 32% | 10 | 34% | 10 | 32% | 9 | 29% | 9 | 23% |
| Breakfast cereals, refined | 2 | 4 | 47% | 4 | 51% | 4 | 58% | 4 | 62% | 2 | -18% |
| **Vegetables** | 157 | 133 | -16% | 137 | -13% | 142 | -9% | 154 | -2% | 175 | 12% |
| **Fruit** |  |  |  |  |  |  |  |  |  |  |  |
| Fruit, other | 55 | 50 | -9% | 52 | -6% | 55 | 1% | 60 | 10% | 65 | 18% |
| Apples and pears | 36 | 41 | 15% | 43 | 19% | 45 | 25% | 48 | 34% | 43 | 18% |
| Citrus fruits | 22 | 18 | -18% | 19 | -14% | 19 | -13% | 21 | -5% | 21 | -2% |
| Berries | 15 | 5 | -65% | 6 | -62% | 7 | -55% | 9 | -42% | 27 | 75% |
| Fruit compote | 4 | 2 | -47% | 2 | -46% | 3 | -40% | 4 | -8% | 10 | 141% |
| Mixed fruits and olives | 3 | 2 | -24% | 2 | -17% | 3 | -7% | 3 | 18% | 5 | 99% |
| **Potatoes and tubers** | 72 | 64 | -10% | 65 | -10% | 66 | -8% | 68 | -5% | 73 | 2% |
| **Nuts and seeds** |  |  |  |  |  |  |  |  |  |  |  |
| Nuts and seeds, not unsalted | 10 | 14 | 46% | 14 | 45% | 13 | 37% | 12 | 28% | 10 | 1% |
| Nuts and seeds, unsalted | 5 | 6 | 12% | 7 | 34% | 9 | 80% | 12 | 142% | 14 | 182% |
| **Legumes** | 7 | 12 | 60% | 15 | 102% | 16 | 119% | 17 | 126% | 16 | 116% |
|  |  |  |  |  |  |  |  |  |  |  |  |
| **ANIMAL-BASED FOOD GROUPS** |  |  |  |  |  |  |  |  |  |  |  |
| **Dairy** |  |  |  |  |  |  |  |  |  |  |  |
| Milk and yoghurt products, sugar free | 232 | 182 | -22% | 185 | -20% | 193 | -17% | 206 | -11% | 223 | -4% |
| Milk and yoghurt products, sugared | 17 | 14 | -19% | 12 | -26% | 10 | -38% | 9 | -48% | 6 | -63% |
| Dairy, other | 60 | 40 | -33% | 40 | -34% | 40 | -34% | 42 | -30% | 58 | -3% |
| **Meat** |  |  |  |  |  |  |  |  |  |  |  |
| Meat, hot meal, processed | 23 | 19 | -21% | 18 | -25% | 16 | -33% | 13 | -44% | 10 | -56% |
| Meat, cold meal, processed | 23 | 19 | -17% | 19 | -17% | 18 | -18% | 17 | -24% | 12 | -46% |
| Meat beef, unprocessed | 15 | 3 | -81% | 3 | -81% | 3 | -80% | 4 | -76% | 14 | -9% |
| Meat poultry, unprocessed | 19 | 21 | 14% | 22 | 14% | 23 | 20% | 25 | 31% | 24 | 28% |
| Meat pork, unprocessed | 12 | 9 | -23% | 9 | -22% | 10 | -16% | 11 | -1% | 14 | 24% |
| Meat, other | 4 | 1 | -67% | 1 | -68% | 1 | -68% | 1 | -65% | 3 | -22% |
| **Cheese** | 35 | 31 | -12% | 31 | -12% | 31 | -11% | 32 | -8% | 34 | -3% |
| **Fish** |  |  |  |  |  |  |  |  |  |  |  |
| Fish, not fatty | 11 | 7 | -34% | 7 | -34% | 7 | -34% | 7 | -31% | 7 | -29% |
| Fish, fatty | 6 | 14 | 123% | 16 | 154% | 19 | 197% | 20 | 212% | 20 | 208% |
| **Eggs** | 18 | 19 | 10% | 19 | 10% | 20 | 13% | 21 | 17% | 21 | 20% |
| **Dairy and meat replacers** |  |  |  |  |  |  |  |  |  |  |  |
| Dairy replacers | 12 | 35 | 188% | 35 | 192% | 35 | 192% | 35 | 187% | 25 | 111% |
| Meat replacers | 3 | 6 | 77% | 6 | 75% | 6 | 73% | 6 | 79% | 5 | 63% |
|  |  |  |  |  |  |  |  |  |  |  |  |
| **MIXED FOOD GROUPS** |  |  |  |  |  |  |  |  |  |  |  |
| **Soups and bouillon** | 18 | 18 | 3% | 19 | 5% | 19 | 7% | 20 | 12% | 20 | 11% |
| **Biscuits and sweet pastries** | 38 | 37 | -2% | 37 | -2% | 38 | -1% | 37 | -2% | 36 | -4% |
| **Contaminants, yeast and sauces** | 35 | 30 | -16% | 30 | -16% | 30 | -16% | 30 | -15% | 31 | -13% |
| **Sugar and confectionary** |  |  |  |  |  |  |  |  |  |  |  |
| Sugar, honey, jam, syrup, dessert sauce | 10 | 8 | -24% | 8 | -24% | 8 | -24% | 8 | -22% | 8 | -18% |
| Chocolate, candy bars, chocolate bread toppings | 10 | 11 | 8% | 12 | 12% | 12 | 14% | 12 | 12% | 10 | -6% |
| Sweets without chocolate, other bread toppings | 4 | 5 | 39% | 5 | 28% | 4 | 16% | 4 | -3% | 4 | 9% |
| **Fats and oils** |  |  |  |  |  |  |  |  |  |  |  |
| Fats and oils, soft | 19 | 18 | -6% | 18 | -5% | 18 | -3% | 19 | -2% | 19 | -1% |
| Fats and oils, hard | 3 | 2 | -36% | 2 | -41% | 2 | -47% | 2 | -50% | 2 | -52% |
| **Savoury snacks** |  |  |  |  |  |  |  |  |  |  |  |
| Pretzels, chips, salty biscuits | 8 | 10 | 24% | 10 | 17% | 9 | 9% | 8 | 1% | 8 | 4% |
| Snacks, croquettes, snack rolls | 11 | 12 | 10% | 12 | 9% | 12 | 8% | 12 | 7% | 12 | 8% |
|  |  |  |  |  |  |  |  |  |  |  |  |
| **BEVERAGES** |  |  |  |  |  |  |  |  |  |  |  |
| **Non-alcoholic beverages** |  |  |  |  |  |  |  |  |  |  |  |
| Coffee and tea | 852 | 620 | -27% | 653 | -23% | 709 | -17% | 809 | -5% | 956 | 12% |
| Water | 927 | 1241 | 34% | 1218 | 31% | 1165 | 26% | 1080 | 16% | 947 | 2% |
| Soft drinks, sugared | 106 | 88 | -16% | 78 | -26% | 68 | -36% | 54 | -49% | 40 | -62% |
| Soft drinks, sugar free | 87 | 45 | -48% | 46 | -47% | 46 | -47% | 48 | -44% | 78 | -10% |
| Alcohol free beverages | 20 | 13 | -31% | 14 | -29% | 14 | -28% | 15 | -25% | 23 | 19% |
| Juice, fruit | 36 | 14 | -61% | 14 | -61% | 14 | -60% | 16 | -56% | 17 | -52% |
| Juice, vegetables | 1 | 0 | -68% | 0 | -65% | 0 | -64% | 0 | -53% | 2 | 98% |
| **Alcoholic beverages** |  |  |  |  |  |  |  |  |  |  |  |
| Beer | 72 | 89 | 23% | 89 | 23% | 88 | 21% | 91 | 26% | 91 | 25% |
| Wine | 42 | 20 | -52% | 20 | -52% | 21 | -50% | 23 | -45% | 40 | -4% |
| Alcohol, other than beer wine | 6 | 8 | 17% | 7 | 8% | 7 | 1% | 6 | -7% | 6 | -5% |

**Supplemental table 3.3.** Daily food consumption in grams and relative (%) difference with current diets, for 669 Dutch adults 18-79 years with high educational level, from the Dutch National Food Consumption Survey 2019-2021.

|  | **Current** | **Model 1** | | **Model 2** | | **Model 3** | | **Model 4** | | **Model 5** | |
| --- | --- | --- | --- | --- | --- | --- | --- | --- | --- | --- | --- |
|  | Mean | Mean |  | Mean |  | Mean |  | Mean |  | Mean |  |
|  | (grams) | (grams) | Δ | (grams) | Δ | (grams) | Δ | (grams) | Δ | (grams) | Δ |
| **PLANT-BASED FOOD GROUPS** | |  |  |  |  |  |  |  |  |  |  |
| **Grains and grain-based products** | |  |  |  |  |  |  |  |  |  |  |
| Bread, rusks, wholegrain | 86 | 98 | 14% | 97 | 12% | 95 | 10% | 92 | 6% | 82 | -5% |
| Bread, rusks, refined | 30 | 23 | -23% | 23 | -25% | 22 | -27% | 21 | -31% | 18 | -42% |
| Flours, rice, grains, refined | 54 | 40 | -26% | 38 | -29% | 36 | -33% | 33 | -39% | 27 | -49% |
| Flours, rice, grains, wholegrain | 13 | 13 | 7% | 14 | 9% | 14 | 13% | 16 | 31% | 26 | 109% |
| Breakfast cereals, wholegrain | 10 | 15 | 45% | 16 | 48% | 16 | 51% | 16 | 56% | 15 | 46% |
| Breakfast cereals, refined | 2 | 2 | -24% | 2 | -23% | 2 | -21% | 2 | -17% | 2 | -30% |
| **Vegetables** | 197 | 186 | -6% | 189 | -4% | 192 | -3% | 197 | 0% | 206 | 5% |
| **Fruit** |  |  |  |  |  |  |  |  |  |  |  |
| Fruit, other | 67 | 67 | 1% | 67 | 1% | 68 | 2% | 71 | 7% | 84 | 26% |
| Apples and pears | 46 | 50 | 7% | 53 | 15% | 56 | 22% | 59 | 27% | 55 | 19% |
| Citrus fruits | 25 | 29 | 16% | 30 | 18% | 30 | 19% | 32 | 25% | 31 | 21% |
| Berries | 19 | 10 | -47% | 10 | -45% | 11 | -42% | 12 | -35% | 22 | 18% |
| Fruit compote | 5 | 3 | -41% | 3 | -40% | 3 | -38% | 3 | -32% | 4 | -23% |
| Mixed fruits and olives | 3 | 4 | 15% | 4 | 15% | 4 | 15% | 4 | 22% | 4 | 12% |
| **Potatoes and tubers** | 64 | 66 | 4% | 67 | 5% | 67 | 5% | 68 | 6% | 65 | 2% |
| **Nuts and seeds** |  |  |  |  |  |  |  |  |  |  |  |
| Nuts and seeds, not unsalted | 11 | 12 | 8% | 11 | 3% | 11 | -1% | 10 | -5% | 10 | -7% |
| Nuts and seeds, unsalted | 8 | 11 | 46% | 12 | 58% | 13 | 71% | 14 | 92% | 16 | 108% |
| **Legumes** | 8 | 9 | 17% | 12 | 53% | 14 | 82% | 15 | 94% | 15 | 99% |
|  |  |  |  |  |  |  |  |  |  |  |  |
| **ANIMAL-BASED FOOD GROUPS** |  |  |  |  |  |  |  |  |  |  |  |
| **Dairy** |  |  |  |  |  |  |  |  |  |  |  |
| Milk and yoghurt products, sugar free | 230 | 199 | -13% | 200 | -13% | 202 | -12% | 205 | -11% | 213 | -7% |
| Milk and yoghurt products, sugared | 12 | 8 | -34% | 8 | -35% | 7 | -37% | 7 | -39% | 5 | -59% |
| Dairy, other | 56 | 48 | -14% | 48 | -14% | 48 | -14% | 51 | -9% | 61 | 9% |
| **Meat** |  |  |  |  |  |  |  |  |  |  |  |
| Meat, hot meal, processed | 19 | 13 | -29% | 13 | -32% | 12 | -36% | 10 | -46% | 8 | -59% |
| Meat, cold meal, processed | 19 | 10 | -46% | 10 | -47% | 10 | -48% | 9 | -51% | 7 | -63% |
| Meat beef, unprocessed | 11 | 4 | -66% | 4 | -65% | 4 | -64% | 4 | -62% | 9 | -23% |
| Meat poultry, unprocessed | 17 | 21 | 23% | 21 | 25% | 22 | 30% | 24 | 44% | 26 | 56% |
| Meat pork, unprocessed | 8 | 7 | -9% | 8 | -8% | 8 | -8% | 8 | -2% | 9 | 7% |
| Meat, other | 4 | 1 | -71% | 1 | -70% | 1 | -70% | 1 | -68% | 3 | -18% |
| **Cheese** | 38 | 35 | -9% | 35 | -9% | 35 | -10% | 34 | -11% | 36 | -7% |
| **Fish** |  |  |  |  |  |  |  |  |  |  |  |
| Fish, not fatty | 11 | 8 | -28% | 8 | -28% | 8 | -28% | 8 | -28% | 8 | -26% |
| Fish, fatty | 9 | 22 | 131% | 23 | 147% | 24 | 159% | 24 | 161% | 22 | 133% |
| **Eggs** | 16 | 15 | -5% | 15 | -5% | 15 | -5% | 15 | -3% | 16 | 0% |
| **Dairy and meat replacers** |  |  |  |  |  |  |  |  |  |  |  |
| Dairy replacers | 14 | 22 | 54% | 21 | 50% | 21 | 46% | 20 | 45% | 25 | 75% |
| Meat replacers | 7 | 10 | 40% | 10 | 36% | 10 | 34% | 10 | 35% | 12 | 63% |
|  |  |  |  |  |  |  |  |  |  |  |  |
| **MIXED FOOD GROUPS** |  |  |  |  |  |  |  |  |  |  |  |
| **Soups and bouillon** | 25 | 25 | -2% | 25 | 1% | 26 | 2% | 26 | 1% | 25 | -1% |
| **Biscuits and sweet pastries** | 40 | 38 | -7% | 38 | -6% | 38 | -6% | 39 | -4% | 40 | -1% |
| **Contaminants, yeast and sauces** | 36 | 33 | -10% | 33 | -10% | 33 | -10% | 33 | -8% | 35 | -2% |
| **Sugar and confectionary** |  |  |  |  |  |  |  |  |  |  |  |
| Sugar, honey, jam, syrup, dessert sauce | 9 | 9 | -1% | 9 | -1% | 9 | -2% | 9 | -4% | 8 | -13% |
| Chocolate, candy bars, chocolate bread toppings | 11 | 11 | -4% | 11 | -7% | 10 | -9% | 10 | -10% | 10 | -9% |
| Sweets without chocolate, other bread toppings | 3 | 4 | 6% | 4 | 14% | 4 | 18% | 4 | 17% | 4 | 26% |
| **Fats and oils** |  |  |  |  |  |  |  |  |  |  |  |
| Fats and oils, soft | 18 | 18 | 2% | 18 | 2% | 18 | 2% | 18 | 2% | 18 | 1% |
| Fats and oils, hard | 5 | 3 | -33% | 3 | -36% | 3 | -38% | 3 | -41% | 3 | -41% |
| **Savoury snacks** |  |  |  |  |  |  |  |  |  |  |  |
| Pretzels, chips, salty biscuits | 8 | 8 | 7% | 8 | 5% | 8 | 1% | 7 | -4% | 7 | -5% |
| Snacks, croquettes, snack rolls | 9 | 9 | 9% | 9 | 8% | 9 | 5% | 9 | 8% | 10 | 20% |
|  |  |  |  |  |  |  |  |  |  |  |  |
| **BEVERAGES** |  |  |  |  |  |  |  |  |  |  |  |
| **Non-alcoholic beverages** |  |  |  |  |  |  |  |  |  |  |  |
| Coffee and tea | 940 | 807 | -14% | 826 | -12% | 854 | -9% | 902 | -4% | 1004 | 7% |
| Water | 881 | 980 | 11% | 966 | 10% | 940 | 7% | 901 | 2% | 828 | -6% |
| Soft drinks, sugared | 82 | 46 | -44% | 45 | -46% | 42 | -49% | 38 | -54% | 33 | -60% |
| Soft drinks, sugar free | 61 | 36 | -42% | 35 | -42% | 36 | -40% | 38 | -37% | 60 | -2% |
| Alcohol free beverages | 21 | 12 | -41% | 13 | -39% | 13 | -37% | 14 | -32% | 19 | -11% |
| Juice, fruit | 33 | 16 | -52% | 15 | -54% | 15 | -55% | 14 | -58% | 13 | -61% |
| Juice, vegetables | 1 | 1 | -42% | 1 | -33% | 1 | -17% | 1 | 7% | 2 | 78% |
| **Alcoholic beverages** |  |  |  |  |  |  |  |  |  |  |  |
| Beer | 69 | 70 | 1% | 69 | 1% | 68 | -1% | 69 | 0% | 75 | 9% |
| Wine | 58 | 36 | -38% | 37 | -36% | 39 | -34% | 41 | -30% | 50 | -13% |
| Alcohol, other than beer wine | 5 | 3 | -36% | 3 | -37% | 3 | -38% | 3 | -37% | 4 | -31% |

## Supplemental file 4. Macro and mirconutrients for current and optimized diets.

**Supplemental table 4.1.** Macro and micronutrient intake of diets for 445 Dutch adults 18-79 years with low educational level, from the Dutch National Food Consumption Survey 2019-2021.

|  | **Observed** | **Model 1** | | **Model 2** | | **Model 3** | | **Model 4** | | **Model 5** | |
| --- | --- | --- | --- | --- | --- | --- | --- | --- | --- | --- | --- |
|  | Mean | Mean |  | Mean |  | Mean |  | Mean |  | Mean |  |
|  | unit | unit | **Δ** | unit | **Δ** | unit | **Δ** | unit | **Δ** | unit | **Δ** |
| Energy (kcal) | 1977 | 1900 | -4% | 1901 | -4% | 1904 | -4% | 1910 | -3% | 1932 | -2% |
| Protein (g) | 80 | 76 | -4% | 77 | -4% | 77 | -4% | 78 | -3% | 79 | -1% |
| Vegetable protein (g) | 29 | 31 | 7% | 31 | 7% | 31 | 6% | 31 | 6% | 30 | 4% |
| Animal protein (g) | 51 | 46 | -11% | 46 | -10% | 46 | -10% | 47 | -8% | 49 | -3% |
| Fat (g) | 84 | 82 | -2% | 82 | -2% | 82 | -2% | 83 | -1% | 84 | 0% |
| SFA (g) | 31 | 28 | -8% | 28 | -9% | 28 | -9% | 28 | -9% | 29 | -6% |
| EPA (g) | 71 | 114 | 60% | 126 | 78% | 138 | 95% | 147 | 107% | 141 | 99% |
| DHA (g) | 107 | 188 | 77% | 211 | 98% | 233 | 119% | 249 | 134% | 236 | 121% |
| Carbohydrates (g) | 202 | 191 | -5% | 191 | -5% | 190 | -6% | 190 | -6% | 189 | -6% |
| Mono and disaccarides (g) | 91 | 85 | -7% | 84 | -7% | 85 | -7% | 85 | -7% | 85 | -6% |
| Dietary fibre (g) | 20 | 21 | 8% | 21 | 9% | 22 | 10% | 22 | 11% | 22 | 12% |
| Alcohol (g) | 8 | 6 | -17% | 7 | -15% | 7 | -13% | 7 | -9% | 8 | 6% |
| Calcium (mg) | 1020 | 969 | -5% | 971 | -5% | 973 | -5% | 983 | -4% | 1010 | -1% |
| Iron (mg) | 10 | 10 | 0% | 10 | 0% | 10 | 0% | 10 | 0% | 10 | 0% |
| Iron haem (mg) | 1 | 0 | -54% | 0 | -53% | 0 | -52% | 1 | -49% | 1 | -26% |
| Iron non-haem (mg) | 9 | 9 | 6% | 9 | 6% | 9 | 6% | 9 | 5% | 9 | 4% |
| Iodine (mg) | 169 | 162 | -4% | 161 | -4% | 160 | -5% | 159 | -6% | 159 | -6% |
| Sodium (mg) | 2285 | 2052 | -10% | 2040 | -11% | 2026 | -11% | 1999 | -13% | 1974 | -14% |
| Zinc (mg) | 10 | 9 | -9% | 9 | -9% | 9 | -9% | 10 | -8% | 10 | -2% |
| ret (ug) | 683 | 673 | -1% | 677 | -1% | 671 | -2% | 644 | -6% | 528 | -23% |
| RAE (ug) | 880 | 904 | 3% | 920 | 5% | 931 | 6% | 915 | 4% | 787 | -11% |
| vitB2 (mg) | 2 | 1 | -7% | 1 | -7% | 1 | -7% | 1 | -7% | 1 | -5% |
| vitB6 (mg) | 2 | 2 | 3% | 2 | 3% | 2 | 4% | 2 | 5% | 2 | 7% |
| vitB12 (ug) | 5 | 4 | -9% | 4 | -7% | 4 | -4% | 5 | -2% | 5 | 2% |

**Supplemental table 4.2.** Macro and micronutrient intake of diets for 663 Dutch adults 18-79 years with intermediate educational level, from the Dutch National Food Consumption Survey 2019-2021.

|  | **Observed** | **Model 1** | | **Model 2** | | **Model 3** | | **Model 4** | | **Model 5** | |
| --- | --- | --- | --- | --- | --- | --- | --- | --- | --- | --- | --- |
|  | Mean | Mean |  | Mean |  | Mean |  | Mean |  | Mean |  |
|  | unit | unit | **Δ** | unit | **Δ** | unit | **Δ** | unit | **Δ** | unit | **Δ** |
| Energy (kcal) | 2061 | 1983 | -4% | 1985 | -4% | 1984 | -4% | 1987 | -4% | 2019 | -2% |
| Protein (g) | 81 | 78 | -4% | 78 | -4% | 78 | -4% | 79 | -3% | 81 | 0% |
| Vegetable protein (g) | 31 | 35 | 12% | 35 | 12% | 34 | 11% | 34 | 9% | 32 | 3% |
| Animal protein (g) | 50 | 43 | -14% | 43 | -14% | 44 | -13% | 45 | -11% | 49 | -2% |
| Fat (g) | 87 | 82 | -6% | 83 | -5% | 84 | -4% | 85 | -3% | 88 | 1% |
| SFA (g) | 31 | 28 | -11% | 28 | -10% | 28 | -9% | 28 | -9% | 29 | -6% |
| EPA (g) | 77 | 124 | 60% | 139 | 79% | 157 | 103% | 166 | 114% | 176 | 127% |
| DHA (g) | 115 | 198 | 72% | 223 | 94% | 254 | 121% | 267 | 131% | 274 | 138% |
| Carbohydrates (g) | 211 | 208 | -2% | 206 | -2% | 204 | -3% | 201 | -5% | 197 | -7% |
| Mono and disaccarides (g) | 91 | 85 | -6% | 85 | -7% | 84 | -7% | 84 | -7% | 86 | -6% |
| Dietary fibre (g) | 21 | 22 | 5% | 22 | 7% | 23 | 8% | 23 | 8% | 22 | 7% |
| Alcohol (g) | 9 | 8 | -10% | 8 | -13% | 7 | -14% | 8 | -13% | 9 | 6% |
| Calcium (mg) | 1005 | 967 | -4% | 965 | -4% | 960 | -5% | 967 | -4% | 1011 | 1% |
| Iron (mg) | 10 | 11 | 7% | 11 | 7% | 11 | 4% | 11 | 1% | 11 | 1% |
| Iron haem (mg) | 1 | 0 | -54% | 0 | -53% | 0 | -50% | 0 | -48% | 1 | -21% |
| Iron non-haem (mg) | 9 | 11 | 13% | 11 | 12% | 10 | 9% | 10 | 5% | 10 | 3% |
| Iodine (mg) | 169 | 167 | -1% | 166 | -2% | 162 | -4% | 157 | -7% | 156 | -8% |
| Sodium (mg) | 2339 | 2252 | -4% | 2222 | -5% | 2179 | -7% | 2122 | -9% | 2025 | -13% |
| Zinc (mg) | 10 | 10 | -7% | 10 | -7% | 9 | -8% | 9 | -8% | 10 | -1% |
| ret (ug) | 570 | 642 | 13% | 636 | 12% | 610 | 7% | 553 | -3% | 501 | -12% |
| RAE (ug) | 778 | 847 | 9% | 856 | 10% | 842 | 8% | 792 | 2% | 732 | -6% |
| vitB2 (mg) | 1 | 1 | 4% | 1 | 3% | 1 | 1% | 1 | -1% | 1 | 1% |
| vitB6 (mg) | 2 | 2 | -1% | 2 | -1% | 2 | -2% | 2 | -1% | 2 | 3% |
| vitB12 (ug) | 4 | 4 | 0% | 4 | 2% | 5 | 4% | 5 | 4% | 5 | 10% |

**Supplemental table 4.3.** Macro and micronutrient intake of diets for 669 Dutch adults 18-79 years with high educational level, from Dutch National Food Consumption Survey 2019-2021.

|  | **Observed** | **Model 1** | | **Model 2** | | **Model 3** | | **Model 4** | | **Model 5** | |
| --- | --- | --- | --- | --- | --- | --- | --- | --- | --- | --- | --- |
|  | Mean | Mean |  | Mean |  | Mean |  | Mean |  | Mean |  |
|  | unit | unit | **Δ** | unit | **Δ** | unit | **Δ** | unit | **Δ** | unit | **Δ** |
| Energy (kcal) | 2111 | 2011 | -5% | 2013 | -5% | 2012 | -5% | 2020 | -4% | 2046 | -3% |
| Protein (g) | 81 | 77 | -5% | 77 | -5% | 77 | -5% | 78 | -4% | 80 | -2% |
| Vegetable protein (g) | 34 | 35 | 4% | 35 | 4% | 35 | 4% | 35 | 3% | 34 | 1% |
| Animal protein (g) | 47 | 41 | -12% | 42 | -11% | 42 | -11% | 43 | -9% | 45 | -3% |
| Fat (g) | 91 | 87 | -4% | 87 | -4% | 87 | -4% | 88 | -3% | 90 | 0% |
| SFA (g) | 32 | 29 | -10% | 29 | -11% | 29 | -11% | 29 | -10% | 30 | -7% |
| EPA (g) | 99 | 191 | 93% | 204 | 107% | 214 | 116% | 217 | 120% | 190 | 92% |
| DHA (g) | 150 | 321 | 114% | 344 | 130% | 361 | 141% | 368 | 146% | 319 | 113% |
| Carbohydrates (g) | 213 | 204 | -4% | 203 | -4% | 202 | -5% | 201 | -5% | 198 | -7% |
| Mono and disaccarides (g) | 92 | 83 | -10% | 83 | -10% | 83 | -10% | 84 | -9% | 87 | -6% |
| Dietary fibre (g) | 23 | 25 | 5% | 25 | 6% | 25 | 7% | 25 | 8% | 25 | 7% |
| Alcohol (g) | 10 | 7 | -25% | 7 | -24% | 8 | -23% | 8 | -21% | 9 | -9% |
| Calcium (mg) | 1040 | 970 | -7% | 969 | -7% | 971 | -7% | 980 | -6% | 1029 | -1% |
| Iron (mg) | 11 | 11 | -2% | 11 | -2% | 11 | -1% | 11 | -1% | 11 | 1% |
| Iron haem (mg) | 1 | 0 | -57% | 0 | -56% | 0 | -56% | 0 | -54% | 0 | -31% |
| Iron non-haem (mg) | 10 | 10 | 2% | 10 | 2% | 10 | 3% | 11 | 3% | 11 | 3% |
| Iodine (mg) | 168 | 163 | -3% | 163 | -3% | 162 | -4% | 161 | -4% | 156 | -7% |
| Sodium (mg) | 2287 | 2163 | -5% | 2150 | -6% | 2126 | -7% | 2083 | -9% | 2014 | -12% |
| Zinc (mg) | 11 | 10 | -10% | 10 | -10% | 10 | -9% | 10 | -9% | 10 | -4% |
| ret (ug) | 647 | 533 | -18% | 539 | -17% | 536 | -17% | 519 | -20% | 510 | -21% |
| RAE (ug) | 916 | 811 | -11% | 817 | -11% | 812 | -11% | 801 | -12% | 795 | -13% |
| vitB2 (mg) | 1 | 1 | -5% | 1 | -5% | 1 | -5% | 1 | -4% | 1 | 0% |
| vitB6 (mg) | 2 | 2 | -1% | 2 | -1% | 2 | 1% | 2 | 2% | 2 | 6% |
| vitB12 (ug) | 5 | 5 | -1% | 5 | 1% | 5 | 3% | 5 | 3% | 5 | 7% |

## Supplemental file 5. Distribution of diet costs for current and optimized diets.

| (a) | 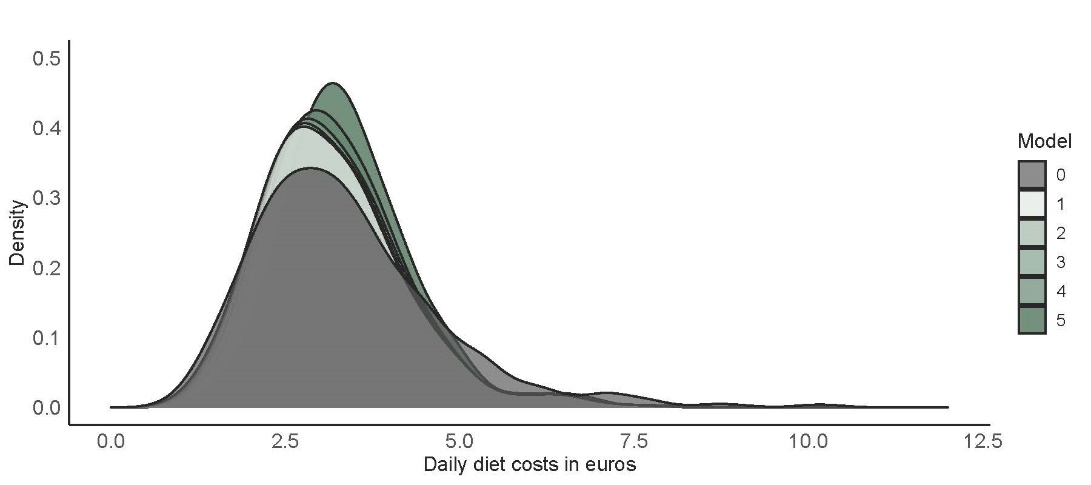 |
| --- | --- |
| (b) | 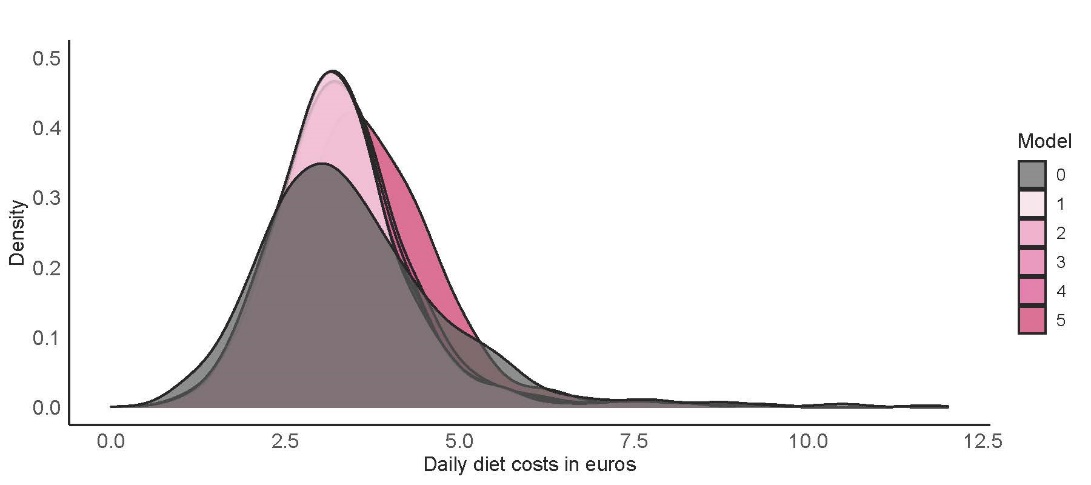 |
| (c) | 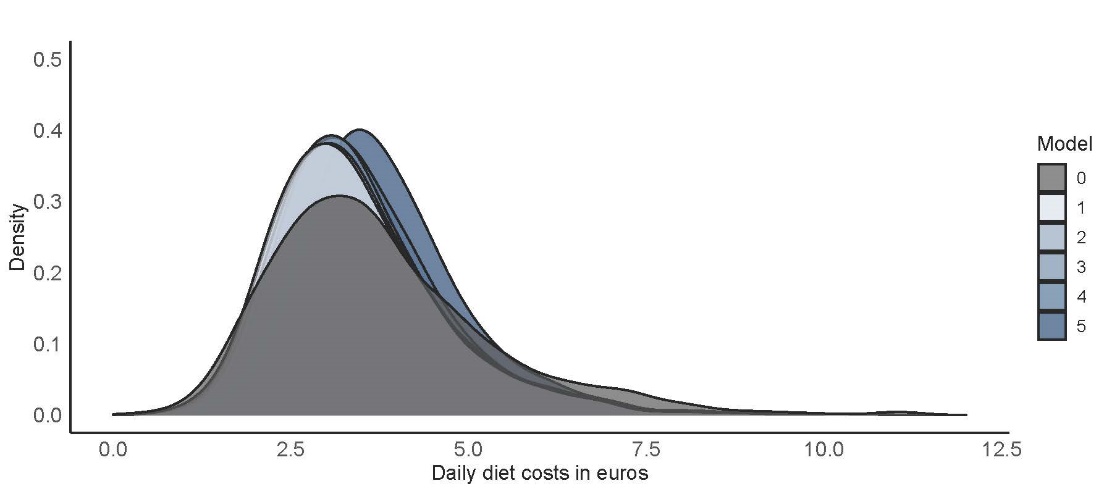 |

**Supplemental figures 5.1a-c**. Distribution of diet costs of current (0) and optimized diets (model 1 to 5) based on minimal costs for Dutch adults aged 18-79 years with low (a), intermediate (b) and high (c) educational level.

## Supplemental file 6. Outcomes diet optimization for secondary analysis, using the total population as peers.

|  |  | | |
| --- | --- | --- | --- |
| (a) | (b) | |  |
|  |  | |  |
| (c) | |  |  |

**Supplemental figures 6.1a-c.** Trade-off between greenhouse gas emissions and Dutch Healthy Diet index 2015 for 1747 Dutch adults aged 18-79 years with low(a), intermediate(b) and high(c) educational level, from the DNFCS 2019-2021, results from the secondary analysis using total population as peers. Dark green circles represent the individual data of current diets and the light green triangles for the optimized diets for all five models (1 to 5). The pink circle indicates the median of current diets, the pink triangles numbered 1 to 5, represent the median of the models, with median prices. This secondary analyses addressed how the stratification for educational peer subgroups influenced the results. Using the total population as peers instead of using educational subgroups as peers, increased the options for making linear combinations of diets. The results consistently showed more pronounced reductions in GHG emissions and increases in DHD15 index for diets of all educational subgroups, while the diet costs were still similar compared to current diets.

**Supplemental table 6.1.** Summary output for characteristics of current and optimized diets for 445 Dutch adults 18-79 years with low educational level, from the Dutch National Food Consumption Survey 2019-2021, results from the secondary analysis using total population as peers.

|  | **Model 1** | | | **Model 2** | | | **Model 3** | | | **Model 4** | | | **Model 5** | | |
| --- | --- | --- | --- | --- | --- | --- | --- | --- | --- | --- | --- | --- | --- | --- | --- |
| Diet costs (€/day) |  |  |  |  |  |  |  |  |  |  |  |  |  |  |  |
| Minimum of minimum price | 3.07 | (2.55 - | 3.72) | 3.09 | (2.56 - | 3.71) | 3.11 | (2.56 - | 3.75) | 3.14 | (2.62 - | 3.78) | 3.39 | (2.87 - | 4.04) |
| Mean of minimum price | 7.59 | (6.42 - | 8.96) | 7.54 | (6.36 - | 8.99) | 7.55 | (6.32 - | 8.98) | 7.48 | (6.30 - | 8.98) | 7.85 | (6.90 - | 9.32) |
| Median of minimum price | 7.33 | (6.20 - | 8.67) | 7.29 | (6.10 - | 8.64) | 7.21 | (6.07 - | 8.57) | 7.14 | (6.02 - | 8.58) | 7.51 | (6.55 - | 8.90) |
|  |  |  |  |  |  |  |  |  |  |  |  |  |  |  |  |
|  |  |  |  |  |  |  |  |  |  |  |  |  |  |  |  |
| DHD15 (points) | 96 | (85 - | 107) | 104 | (93 - | 113) | 112 | (102 - | 120) | 121 | (110 - | 128) | 130 | (118 - | 136) |
| Energy (kcal) | 1908 | (599) |  | 1910 | (600) |  | 1914 | (599) |  | 1921 | (596) |  | 1953 | (591) |  |
| Protein (g) | 77 | (26) |  | 77 | (26) |  | 77 | (26) |  | 78 | (26) |  | 80 | (26) |  |
| Plant protein (%) | 43% |  |  | 43% |  |  | 43% |  |  | 41% |  |  | 39% |  |  |
|  |  |  |  |  |  |  |  |  |  |  |  |  |  |  |  |
| GHG emissions (kg CO_2_-eq/d) | 3.26 | (2.72 - | 4.17) | 3.28 | (2.73 - | 4.19) | 3.33 | (2.77 - | 4.24) | 3.48 | (2.87 - | 4.37) | 4.30 | (3.49 - | 5.33) |
| Land use (m^2^*yr/d) | 2.26 | (1.87 - | 2.80) | 2.25 | (1.88 - | 2.82) | 2.29 | (1.91 - | 2.86) | 2.36 | (1.98 - | 2.93) | 2.67 | (2.23 - | 3.26) |
| Freshwater eutrophication (g P-eq/d) | 0.28 | (0.23 - | 0.34) | 0.28 | (0.23 - | 0.35) | 0.28 | (0.23 - | 0.35) | 0.29 | (0.24 - | 0.36) | 0.32 | (0.27 - | 0.40) |
| Marine water eutrophication (g N-eq/d) | 4.76 | (3.95 - | 6.01) | 4.79 | (3.97 - | 6.03) | 4.88 | (4.03 - | 6.11) | 5.02 | (4.15 - | 6.21) | 6.31 | (5.03 - | 8.11) |
| Acidification (g SO_2_-eq/d) | 27.89 | (22.68 - | 35.88) | 27.85 | (22.84 - | 36.10) | 28.34 | (23.15 - | 36.50) | 29.42 | (23.89 - | 37.51) | 37.98 | (29.35 - | 48.98) |
| Blue water (m^3^/d) | 0.13 | (0.10 - | 0.17) | 0.14 | (0.11 - | 0.17) | 0.15 | (0.12 - | 0.18) | 0.16 | (0.13 - | 0.19) | 0.19 | (0.15 - | 0.22) |
|  |  |  |  |  |  |  |  |  |  |  |  |  |  |  |  |
| PREF | 858 | (657 - | 1057) | 855 | (662 - | 1058) | 870 | (686 - | 1065) | 901 | (715 - | 1099) | 954 | (766 - | 1177) |
|  |  |  |  |  |  |  |  |  |  |  |  |  |  |  |  |

*Values are presented as proportions, means (SD) and medians (25^th-^75^th^ percentile).*

*DHD15, Dutch Healthy Diet 2015 index ; PREF, preferences score based on deviation from current diets*

**Supplemental Table 6.2.** Summary output for characteristics of current and optimized diets for 663 Dutch adults 18-79 years with intermediate educational level, from the Dutch National Food Consumption Survey 2019-2021, results from the secondary analysis using total population as peers.

|  | **Model 1** | | | **Model 2** | | | **Model 3** | | | **Model 4** | | | **Model 5** | | |
| --- | --- | --- | --- | --- | --- | --- | --- | --- | --- | --- | --- | --- | --- | --- | --- |
| Diet costs (€/day) |  |  |  |  |  |  |  |  |  |  |  |  |  |  |  |
| Minimum of minimum price | 3.20 | (2.61 - | 3.82) | 3.21 | (2.64 - | 3.82) | 3.22 | (2.66 - | 3.84) | 3.25 | (2.71 - | 3.89) | 3.46 | (2.94 - | 4.15) |
| Mean of minimum price | 7.84 | (6.57 - | 9.19) | 7.84 | (6.55 - | 9.20) | 7.81 | (6.49 - | 9.20) | 7.76 | (6.50 - | 9.19) | 8.16 | (6.99 - | 9.73) |
| Median of minimum price | 7.58 | (6.33 - | 8.83) | 7.54 | (6.28 - | 8.82) | 7.51 | (6.25 - | 8.80) | 7.44 | (6.24 - | 8.80) | 7.77 | (6.68 - | 9.25) |
|  |  |  |  |  |  |  |  |  |  |  |  |  |  |  |  |
|  |  |  |  |  |  |  |  |  |  |  |  |  |  |  |  |
| DHD15 (points) | 95 | (85 - | 107) | 103 | (93 - | 113) | 111 | (101 - | 120) | 119 | (109 - | 128) | 128 | (116 - | 136) |
| Energy (kcal) | 1989 | (666) |  | 1991 | (665) |  | 1995 | (663) |  | 2001 | (659) |  | 2032 | (652) |  |
| Protein (g) | 78 | (29) |  | 79 | (29) |  | 79 | (29) |  | 79 | (29) |  | 81 | (29) |  |
| Plant protein (%) | 44% |  |  | 44% |  |  | 43% |  |  | 43% |  |  | 41% |  |  |
|  |  |  |  |  |  |  |  |  |  |  |  |  |  |  |  |
| GHG emissions (kg CO_2_-eq/d) | 3.39 | (2.69 - | 4.34) | 3.41 | (2.70 - | 4.36) | 3.45 | (2.75 - | 4.41) | 3.56 | (2.85 - | 4.56) | 4.30 | (3.48 - | 5.39) |
| Land use (m^2^*yr/d) | 2.33 | (1.85 - | 2.88) | 2.33 | (1.88 - | 2.89) | 2.35 | (1.90 - | 2.92) | 2.44 | (1.97 - | 3.01) | 2.72 | (2.20 - | 3.34) |
| Freshwater eutrophication (g P-eq/d) | 0.29 | (0.23 - | 0.35) | 0.29 | (0.23 - | 0.36) | 0.29 | (0.24 - | 0.36) | 0.30 | (0.25 - | 0.37) | 0.33 | (0.27 - | 0.41) |
| Marine water eutrophication (g N-eq/d) | 4.91 | (3.91 - | 6.25) | 4.92 | (3.91 - | 6.30) | 4.97 | (3.94 - | 6.33) | 5.05 | (4.08 - | 6.52) | 6.33 | (4.93 - | 8.10) |
| Acidification (g SO_2_-eq/d) | 28.16 | (21.74 - | 36.72) | 28.31 | (21.88 - | 37.17) | 28.48 | (22.17 - | 37.45) | 29.28 | (23.05 - | 38.65) | 37.43 | (28.62 - | 48.68) |
| Blue water (m^3^/d) | 0.14 | (0.11 - | 0.18) | 0.15 | (0.12 - | 0.19) | 0.16 | (0.13 - | 0.19) | 0.17 | (0.14 - | 0.20) | 0.19 | (0.16 - | 0.23) |
|  |  |  |  |  |  |  |  |  |  |  | - |  |  |  |  |
| PREF | 865 | (639 - | 1095) | 877 | (651 - | 1106) | 873 | (684 - | 1112) | 925 | (709 | 1157) | 975 | (761 - | 1233) |
|  |  |  |  |  |  |  |  |  |  |  |  |  |  |  |  |

*Values are presented as proportions, means (SD) and medians (25^th-^75^th^ percentile).*

*DHD15, Dutch Healthy Diet 2015 index; PREF, preferences score based on deviation from current diets*

**Supplemental table 6.3.** Summary output for characteristics of current and optimized diets for 669 Dutch adults 18-79 years with high educational level, from Dutch National Food Consumption Survey 2019-2021 , results from the secondary analysis using total population as peers.

|  | **Model 1** | | | **Model 2** | | | **Model 3** | | | **Model 4** | | | **Model 5** | | |
| --- | --- | --- | --- | --- | --- | --- | --- | --- | --- | --- | --- | --- | --- | --- | --- |
| Diet costs (€/day) |  |  |  |  |  |  |  |  |  |  |  |  |  |  |  |
| Minimum of minimum price | 3.25 | (2.68 - | 3.95) | 3.28 | (2.73 - | 3.94) | 3.30 | (2.75 - | 3.98) | 3.35 | (2.76 - | 3.99) | 3.56 | (3.05 - | 4.26) |
| Mean of minimum price | 8.07 | (6.87 - | 9.58) | 8.08 | (6.84 - | 9.55) | 8.02 | (6.83 - | 9.55) | 8.02 | (6.78 - | 9.59) | 8.44 | (7.27 - | 10.06) |
| Median of minimum price | 7.74 | (6.61 - | 9.15) | 7.74 | (6.60 - | 9.14) | 7.70 | (6.57 - | 9.13) | 7.68 | (6.50 - | 9.14) | 8.07 | (6.93 - | 9.54) |
|  |  |  |  |  |  |  |  |  |  |  |  |  |  |  |  |
|  |  |  |  |  |  |  |  |  |  |  |  |  |  |  |  |
| DHD15 (points) | 100 | (88 - | 111) | 107 | (96 - | 117) | 114 | (104 - | 122) | 122 | (111 - | 129) | 130 | (118 - | 136) |
| Energy (kcal) | 2032 | (654) |  | 2033 | (653) |  | 2036 | (652) |  | 2042 | (648) |  | 2069 | (639) |  |
| Protein (g) | 78 | (26) |  | 78 | (26) |  | 78 | (26) |  | 79 | (26) |  | 81 | (26) |  |
| Plant protein (%) | 46% |  |  | 46% |  |  | 46% |  |  | 46% |  |  | 43% |  |  |
|  |  |  |  |  |  |  |  |  |  |  |  |  |  |  |  |
| GHG emissions (kg CO_2_-eq/d) | 3.37 | (2.77 - | 4.37) | 3.40 | (2.78 - | 4.39) | 3.45 | (2.83 - | 4.46) | 3.56 | (2.92 - | 4.61) | 4.40 | (3.45 - | 5.40) |
| Land use (m^2^*yr/d) | 2.33 | (1.90 - | 2.88) | 2.35 | (1.92 - | 2.88) | 2.38 | (1.94 - | 2.92) | 2.44 | (1.99 - | 3.00) | 2.73 | (2.19 - | 3.30) |
| Freshwater eutrophication (g P-eq/d) | 0.29 | (0.24 - | 0.36) | 0.29 | (0.24 - | 0.36) | 0.30 | (0.24 - | 0.36) | 0.30 | (0.25 - | 0.37) | 0.33 | (0.27 - | 0.41) |
| Marine water eutrophication (g N-eq/d) | 4.86 | (4.02 - | 6.23) | 4.88 | (4.03 - | 6.22) | 4.91 | (4.07 - | 6.30) | 5.05 | (4.18 - | 6.44) | 6.21 | (4.84 - | 8.06) |
| Acidification (g SO_2_-eq/d) | 27.71 | (21.99 - | 36.47) | 27.69 | (22.12 - | 36.63) | 28.10 | (22.44 - | 37.11) | 28.86 | (23.15 - | 38.27) | 36.46 | (27.54 - | 47.84) |
| Blue water (m^3^/d) | 0.16 | (0.12 - | 0.21) | 0.16 | (0.13 - | 0.21) | 0.17 | (0.14 - | 0.21) | 0.18 | (0.15 - | 0.22) | 0.20 | (0.17 - | 0.24) |
|  |  |  |  |  |  |  |  |  |  |  |  |  |  |  |  |
| PREF | 867 | (668 - | 1082) | 875 | (676 - | 1089) | 893 | (695 - | 1120) | 935 | (719 - | 1152) | 992 | (779 - | 1229) |
|  |  |  |  |  |  |  |  |  |  |  |  |  |  |  |  |

*Values are presented as proportions, means (SD) and medians (25^th-^75^th^ percentile).*

*DHD15, Dutch Healthy Diet 2015 index; PREF, preferences score based on deviation from current diets*

**Supplemental table 6.4**. Daily food consumption in grams and relative (%) difference with current diets, for 445 Dutch adults 18-79 years with low educational level, from the Dutch National Food Consumption Survey 2019-2021, results from the secondary analysis using total population as peers.

|  | **Current** | **Model 1** | | **Model 2** | | **Model 3** | | **Model 4** | | **Model 5** | |
| --- | --- | --- | --- | --- | --- | --- | --- | --- | --- | --- | --- |
|  | Mean | Mean |  | Mean |  | Mean |  | Mean |  | Mean |  |
|  | (grams) | (grams) | Δ | (grams) | Δ | (grams) | Δ | (grams) | Δ | (grams) | Δ |
| **PLANT-BASED FOOD GROUPS** | | |  |  |  |  |  |  |  |  |  |
| **Grains and grain-based products** | | |  |  |  |  |  |  |  |  |  |
| Bread, rusks, wholegrain | 79 | 91 | 16% | 91 | 16% | 91 | 15% | 89 | 14% | 81 | 3% |
| Bread, rusks, refined | 35 | 23 | -35% | 22 | -38% | 21 | -41% | 18 | -47% | 14 | -60% |
| Flours, rice, grains, refined | 40 | 26 | -35% | 25 | -38% | 23 | -42% | 21 | -48% | 16 | -60% |
| Flours, rice, grains, wholegrain | 7 | 9 | 34% | 10 | 43% | 11 | 60% | 13 | 99% | 24 | 256% |
| Breakfast cereals, wholegrain | 5 | 12 | 125% | 12 | 126% | 12 | 137% | 14 | 163% | 13 | 161% |
| Breakfast cereals, refined | 2 | 2 | 26% | 2 | 23% | 2 | 24% | 2 | 30% | 2 | -12% |
| **Vegetables** | 150 | 151 | 0% | 154 | 2% | 159 | 6% | 167 | 11% | 181 | 20% |
| **Fruit** |  |  |  |  |  |  |  |  |  |  |  |
| Fruit, other | 50 | 56 | 12% | 57 | 14% | 59 | 18% | 65 | 30% | 74 | 49% |
| Apples and pears | 35 | 46 | 31% | 48 | 38% | 51 | 46% | 54 | 55% | 52 | 49% |
| Citrus fruits | 27 | 25 | -10% | 25 | -8% | 26 | -4% | 29 | 5% | 28 | 1% |
| Berries | 11 | 5 | -52% | 6 | -50% | 6 | -47% | 7 | -39% | 19 | 72% |
| Fruit compote | 5 | 3 | -46% | 3 | -46% | 3 | -44% | 3 | -36% | 6 | 11% |
| Mixed fruits and olives | 3 | 3 | 0% | 3 | 9% | 3 | 18% | 4 | 33% | 5 | 87% |
| **Potatoes and tubers** | 76 | 74 | -2% | 75 | -1% | 75 | 0% | 76 | 1% | 76 | 1% |
| **Nuts and seeds** |  |  |  |  |  |  |  |  |  |  |  |
| Nuts and seeds, not unsalted | 6 | 10 | 57% | 10 | 53% | 9 | 44% | 9 | 37% | 9 | 36% |
| Nuts and seeds, unsalted | 5 | 10 | 102% | 11 | 124% | 13 | 162% | 15 | 199% | 15 | 206% |
| **Legumes** | 7 | 9 | 36% | 13 | 89% | 15 | 123% | 15 | 130% | 16 | 134% |
|  |  |  |  |  |  |  |  |  |  |  |  |
| **ANIMAL-BASED FOOD GROUPS** |  |  |  |  |  |  |  |  |  |  |  |
| **Dairy** |  |  |  |  |  |  |  |  |  |  |  |
| Milk and yoghurt products, sugar free | 263 | 224 | -15% | 225 | -14% | 227 | -14% | 231 | -12% | 247 | -6% |
| Milk and yoghurt products, sugared | 16 | 8 | -51% | 7 | -56% | 6 | -61% | 5 | -68% | 3 | -80% |
| Dairy, other | 69 | 51 | -25% | 51 | -25% | 52 | -25% | 54 | -22% | 58 | -15% |
| **Meat** |  |  |  |  |  |  |  |  |  |  |  |
| Meat, hot meal, processed | 30 | 21 | -31% | 20 | -33% | 19 | -38% | 14 | -52% | 7 | -75% |
| Meat, cold meal, processed | 24 | 14 | -40% | 14 | -41% | 13 | -43% | 11 | -52% | 6 | -73% |
| Meat beef, unprocessed | 17 | 3 | -83% | 3 | -83% | 3 | -82% | 3 | -80% | 11 | -32% |
| Meat poultry, unprocessed | 16 | 23 | 39% | 23 | 41% | 25 | 51% | 30 | 82% | 33 | 104% |
| Meat pork, unprocessed | 14 | 12 | -15% | 12 | -14% | 12 | -13% | 14 | -3% | 14 | 1% |
| Meat, other | 3 | 1 | -68% | 1 | -64% | 1 | -63% | 2 | -55% | 4 | 23% |
| **Cheese** | 32 | 28 | -11% | 28 | -11% | 28 | -11% | 28 | -11% | 30 | -5% |
| **Fish** |  |  |  |  |  |  |  |  |  |  |  |
| Fish, not fatty | 9 | 5 | -43% | 5 | -44% | 5 | -46% | 5 | -44% | 6 | -37% |
| Fish, fatty | 6 | 18 | 222% | 20 | 255% | 22 | 284% | 22 | 289% | 20 | 256% |
| **Eggs** | 16 | 16 | 0% | 16 | 0% | 16 | 1% | 17 | 5% | 19 | 19% |
| **Dairy and meat replacers** | |  |  |  |  |  |  |  |  |  |  |
| Dairy replacers | 6 | 22 | 240% | 22 | 245% | 22 | 239% | 20 | 217% | 20 | 213% |
| Meat replacers | 3 | 6 | 98% | 5 | 87% | 5 | 80% | 5 | 87% | 6 | 112% |
|  |  |  |  |  |  |  |  |  |  |  |  |
| **MIXED FOOD GROUPS** |  |  |  |  |  |  |  |  |  |  |  |
| **Soups and bouillon** | 22 | 22 | 0% | 23 | 5% | 24 | 7% | 25 | 12% | 25 | 14% |
| **Biscuits and sweet pastries** | 42 | 38 | -10% | 38 | -10% | 38 | -11% | 38 | -9% | 40 | -6% |
| **Contaminants, yeast and sauces** | 34 | 30 | -13% | 30 | -12% | 31 | -11% | 31 | -10% | 32 | -7% |
| **Sugar and confectionary** | |  |  |  |  |  |  |  |  |  |  |
| Sugar, honey, jam, syrup, dessert sauce | 11 | 9 | -15% | 9 | -14% | 9 | -15% | 9 | -15% | 9 | -21% |
| Chocolate, candy bars, chocolate bread toppings | 8 | 9 | 18% | 9 | 16% | 9 | 15% | 9 | 19% | 10 | 24% |
| Sweets without chocolate, other bread toppings | 2 | 4 | 68% | 4 | 68% | 4 | 64% | 4 | 46% | 4 | 44% |
| **Fats and oils** |  |  |  |  |  |  |  |  |  |  |  |
| Fats and oils, soft | 19 | 19 | -1% | 19 | -1% | 19 | 0% | 19 | 0% | 19 | 1% |
| Fats and oils, hard | 4 | 2 | -50% | 2 | -51% | 2 | -53% | 2 | -54% | 2 | -55% |
| **Savoury snacks** |  |  |  |  |  |  |  |  |  |  |  |
| Pretzels, chips, salty biscuits | 6 | 7 | 16% | 7 | 13% | 6 | 5% | 6 | 3% | 6 | -4% |
| Snacks, croquettes, snack rolls | 6 | 8 | 23% | 8 | 21% | 8 | 21% | 8 | 22% | 9 | 39% |
|  |  |  |  |  |  |  |  |  |  |  |  |
| **BEVERAGES** |  |  |  |  |  |  |  |  |  |  |  |
| **Non-alcoholic beverages** | |  |  |  |  |  |  |  |  |  |  |
| Coffee and tea | 898 | 727 | -19% | 748 | -17% | 782 | -13% | 844 | -6% | 931 | 4% |
| Water | 713 | 1051 | 47% | 1032 | 45% | 1011 | 42% | 956 | 34% | 846 | 19% |
| Soft drinks, sugared | 121 | 50 | -59% | 47 | -61% | 42 | -65% | 37 | -70% | 29 | -76% |
| Soft drinks, sugar free | 61 | 28 | -55% | 29 | -53% | 29 | -52% | 32 | -47% | 60 | -3% |
| Alcohol free beverages | 17 | 12 | -28% | 13 | -24% | 13 | -23% | 14 | -16% | 19 | 8% |
| Juice, fruit | 25 | 7 | -71% | 7 | -73% | 6 | -74% | 6 | -76% | 5 | -79% |
| Juice, vegetables | 2 | 1 | -65% | 1 | -63% | 1 | -62% | 1 | -54% | 2 | 20% |
| **Alcoholic beverages** |  |  |  |  |  |  |  |  |  |  |  |
| Beer | 66 | 81 | 23% | 80 | 21% | 81 | 22% | 80 | 22% | 78 | 18% |
| Wine | 34 | 16 | -54% | 16 | -53% | 17 | -49% | 19 | -42% | 35 | 4% |
| Alcohol, other than beer wine | 6 | 4 | -32% | 4 | -33% | 4 | -36% | 4 | -39% | 5 | -27% |

**Supplemental table 6.5**. Daily food consumption in grams and relative (%) difference with current diets, for 663 Dutch adults 18-79 years with intermediate educational level, from the Dutch National Food Consumption Survey 2019-2021, results from the secondary analysis using total population as peers.

|  | **Current** | **Model 1** | | **Model 2** | | **Model 3** | | **Model 4** | | **Model 5** | |
| --- | --- | --- | --- | --- | --- | --- | --- | --- | --- | --- | --- |
|  | Mean | Mean |  | Mean |  | Mean |  | Mean |  | Mean |  |
|  | (grams) | (grams) | Δ | (grams) | Δ | (grams) | Δ | (grams) | Δ | (grams) | Δ |
| **PLANT-BASED FOOD GROUPS** | | |  |  |  |  |  |  |  |  |  |
| **Grains and grain-based products** |  |  |  |  |  |  |  |  |  |  |  |
| Bread, rusks, wholegrain | 83 | 91 | 10% | 91 | 10% | 91 | 9% | 89 | 8% | 81 | -2% |
| Bread, rusks, refined | 37 | 23 | -38% | 22 | -41% | 21 | -44% | 18 | -50% | 14 | -62% |
| Flours, rice, grains, refined | 50 | 26 | -48% | 25 | -51% | 23 | -54% | 21 | -59% | 16 | -68% |
| Flours, rice, grains, wholegrain | 8 | 9 | 9% | 10 | 16% | 11 | 30% | 13 | 62% | 24 | 189% |
| Breakfast cereals, wholegrain | 7 | 12 | 58% | 12 | 58% | 12 | 66% | 14 | 84% | 13 | 83% |
| Breakfast cereals, refined | 2 | 2 | -4% | 2 | -6% | 2 | -5% | 2 | -1% | 2 | -33% |
| **Vegetables** | 157 | 151 | -4% | 154 | -2% | 159 | 1% | 167 | 6% | 181 | 15% |
| **Fruit** |  |  |  |  |  |  |  |  |  |  |  |
| Fruit, other | 55 | 56 | 1% | 57 | 3% | 59 | 6% | 65 | 18% | 74 | 34% |
| Apples and pears | 36 | 46 | 27% | 48 | 33% | 51 | 42% | 54 | 51% | 52 | 45% |
| Citrus fruits | 22 | 25 | 13% | 25 | 15% | 26 | 21% | 29 | 32% | 28 | 27% |
| Berries | 15 | 5 | -64% | 6 | -63% | 6 | -61% | 7 | -55% | 19 | 27% |
| Fruit compote | 4 | 3 | -35% | 3 | -34% | 3 | -32% | 3 | -22% | 6 | 34% |
| Mixed fruits and olives | 3 | 3 | 1% | 3 | 10% | 3 | 19% | 4 | 34% | 5 | 89% |
| **Potatoes and tubers** | 72 | 74 | 3% | 75 | 4% | 75 | 5% | 76 | 6% | 76 | 6% |
| **Nuts and seeds** |  |  |  |  |  |  |  |  |  |  |  |
| Nuts and seeds, not unsalted | 10 | 10 | 2% | 10 | 0% | 9 | -6% | 9 | -11% | 9 | -11% |
| Nuts and seeds, unsalted | 5 | 10 | 95% | 11 | 116% | 13 | 153% | 15 | 188% | 15 | 195% |
| **Legumes** | 7 | 9 | 24% | 13 | 72% | 15 | 102% | 15 | 109% | 16 | 113% |
|  |  |  |  |  |  |  |  |  |  |  |  |
| **ANIMAL-BASED FOOD GROUPS** |  |  |  |  |  |  |  |  |  |  |  |
| **Dairy** |  |  |  |  |  |  |  |  |  |  |  |
| Milk and yoghurt products, sugar free | 232 | 224 | -4% | 225 | -3% | 227 | -2% | 231 | -1% | 247 | 6% |
| Milk and yoghurt products, sugared | 17 | 8 | -55% | 7 | -59% | 6 | -63% | 5 | -70% | 3 | -81% |
| Dairy, other | 60 | 51 | -14% | 51 | -15% | 52 | -14% | 54 | -10% | 58 | -3% |
| **Meat** |  |  |  |  |  |  |  |  |  |  |  |
| Meat, hot meal, processed | 23 | 21 | -10% | 20 | -13% | 19 | -19% | 14 | -38% | 7 | -68% |
| Meat, cold meal, processed | 23 | 14 | -37% | 14 | -38% | 13 | -40% | 11 | -50% | 6 | -72% |
| Meat beef, unprocessed | 15 | 3 | -81% | 3 | -81% | 3 | -80% | 3 | -77% | 11 | -24% |
| Meat poultry, unprocessed | 19 | 23 | 21% | 23 | 22% | 25 | 31% | 30 | 58% | 33 | 77% |
| Meat pork, unprocessed | 12 | 12 | 4% | 12 | 5% | 12 | 7% | 14 | 19% | 14 | 24% |
| Meat, other | 4 | 1 | -72% | 1 | -68% | 1 | -67% | 2 | -61% | 4 | 8% |
| **Cheese** | 35 | 28 | -20% | 28 | -20% | 28 | -20% | 28 | -19% | 30 | -14% |
| **Fish** |  |  |  |  |  |  |  |  |  |  |  |
| Fish, not fatty | 11 | 5 | -50% | 5 | -50% | 5 | -52% | 5 | -51% | 6 | -44% |
| Fish, fatty | 6 | 18 | 190% | 20 | 220% | 22 | 245% | 22 | 250% | 20 | 220% |
| **Eggs** | 18 | 16 | -9% | 16 | -10% | 16 | -8% | 17 | -4% | 19 | 8% |
| **Dairy and meat replacers** | |  |  |  |  |  |  |  |  |  |  |
| Dairy replacers | 12 | 22 | 81% | 22 | 84% | 22 | 80% | 20 | 69% | 20 | 66% |
| Meat replacers | 3 | 6 | 72% | 5 | 62% | 5 | 57% | 5 | 63% | 6 | 84% |
|  |  |  |  |  |  |  |  |  |  |  |  |
| **MIXED FOOD GROUPS** |  |  |  |  |  |  |  |  |  |  |  |
| **Soups and bouillon** | 18 | 22 | 25% | 23 | 31% | 24 | 34% | 25 | 40% | 25 | 43% |
| **Biscuits and sweet pastries** | 38 | 38 | 0% | 38 | -1% | 38 | -1% | 38 | 0% | 40 | 5% |
| **Contaminants, yeast and sauces** | 35 | 30 | -15% | 30 | -14% | 31 | -13% | 31 | -12% | 32 | -9% |
| **Sugar and confectionary** | |  |  |  |  |  |  |  |  |  |  |
| Sugar, honey, jam, syrup, dessert sauce | 10 | 9 | -9% | 9 | -8% | 9 | -9% | 9 | -9% | 9 | -15% |
| Chocolate, candy bars, chocolate bread toppings | 10 | 9 | -11% | 9 | -13% | 9 | -14% | 9 | -11% | 10 | -7% |
| Sweets without chocolate, other bread toppings | 4 | 4 | 8% | 4 | 9% | 4 | 6% | 4 | -6% | 4 | -7% |
| **Fats and oils** |  |  |  |  |  |  |  |  |  |  |  |
| Fats and oils, soft | 19 | 19 | -2% | 19 | -2% | 19 | -2% | 19 | -2% | 19 | 0% |
| Fats and oils, hard | 3 | 2 | -38% | 2 | -40% | 2 | -42% | 2 | -44% | 2 | -44% |
| **Savoury snacks** |  |  |  |  |  |  |  |  |  |  |  |
| Pretzels, chips, salty biscuits | 8 | 7 | -15% | 7 | -17% | 6 | -23% | 6 | -24% | 6 | -29% |
| Snacks, croquettes, snack rolls | 11 | 8 | -29% | 8 | -30% | 8 | -30% | 8 | -30% | 9 | -20% |
|  |  |  |  |  |  |  |  |  |  |  |  |
| **BEVERAGES** |  |  |  |  |  |  |  |  |  |  |  |
| **Non-alcoholic beverages** | |  |  |  |  |  |  |  |  |  |  |
| Coffee and tea | 852 | 727 | -15% | 748 | -12% | 782 | -8% | 844 | -1% | 931 | 9% |
| Water | 927 | 1051 | 13% | 1032 | 11% | 1011 | 9% | 956 | 3% | 846 | -9% |
| Soft drinks, sugared | 106 | 50 | -53% | 47 | -55% | 42 | -60% | 37 | -65% | 29 | -73% |
| Soft drinks, sugar free | 87 | 28 | -68% | 29 | -67% | 29 | -66% | 32 | -63% | 60 | -31% |
| Alcohol free beverages | 20 | 12 | -36% | 13 | -33% | 13 | -32% | 14 | -26% | 19 | -5% |
| Juice, fruit | 36 | 7 | -80% | 7 | -81% | 6 | -82% | 6 | -84% | 5 | -85% |
| Juice, vegetables | 1 | 1 | -27% | 1 | -24% | 1 | -22% | 1 | -5% | 2 | 148% |
| **Alcoholic beverages** |  |  |  |  |  |  |  |  |  |  |  |
| Beer | 72 | 81 | 12% | 80 | 11% | 81 | 11% | 80 | 11% | 78 | 8% |
| Wine | 42 | 16 | -63% | 16 | -62% | 17 | -59% | 19 | -53% | 35 | -16% |
| Alcohol, other than beer wine | 6 | 4 | -34% | 4 | -35% | 4 | -38% | 4 | -40% | 5 | -29% |

**Supplemental table 6.6**. Daily food consumption in grams and relative (%) difference with current diets, for 669 Dutch adults 18-79 years with high educational level, from Dutch National Food Consumption Survey 2019-2021, results from the secondary analysis using total population as peers.

|  | **Current** | **Model 1** | | **Model 2** | | **Model 3** | | **Model 4** | | **Model 5** | |
| --- | --- | --- | --- | --- | --- | --- | --- | --- | --- | --- | --- |
|  | Mean | Mean |  | Mean |  | Mean |  | Mean |  | Mean |  |
|  | (grams) | (grams) | Δ | (grams) | Δ | (grams) | Δ | (grams) | Δ | (grams) | Δ |
| **PLANT-BASED FOOD GROUPS** | | |  |  |  |  |  |  |  |  |  |
| **Grains and grain-based products** |  |  |  |  |  |  |  |  |  |  |  |
| Bread, rusks, wholegrain | 86 | 92 | 7% | 91 | 6% | 91 | 6% | 90 | 5% | 83 | -3% |
| Bread, rusks, refined | 30 | 25 | -17% | 24 | -19% | 23 | -23% | 21 | -29% | 17 | -45% |
| Flours, rice, grains, refined | 54 | 35 | -36% | 33 | -39% | 32 | -42% | 28 | -48% | 23 | -57% |
| Flours, rice, grains, wholegrain | 13 | 10 | -17% | 11 | -12% | 12 | -2% | 15 | 20% | 25 | 100% |
| Breakfast cereals, wholegrain | 10 | 13 | 23% | 13 | 24% | 13 | 26% | 14 | 35% | 14 | 31% |
| Breakfast cereals, refined | 2 | 3 | 14% | 3 | 15% | 3 | 13% | 3 | 8% | 2 | -33% |
| **Vegetables** | 197 | 154 | -22% | 156 | -21% | 160 | -19% | 167 | -15% | 179 | -9% |
| **Fruit** |  |  |  |  |  |  |  |  |  |  |  |
| Fruit, other | 67 | 58 | -14% | 58 | -12% | 61 | -9% | 65 | -3% | 75 | 12% |
| Apples and pears | 46 | 46 | 1% | 49 | 6% | 52 | 12% | 54 | 18% | 52 | 14% |
| Citrus fruits | 25 | 24 | -7% | 24 | -6% | 25 | -3% | 27 | 6% | 26 | 3% |
| Berries | 19 | 6 | -70% | 6 | -69% | 6 | -67% | 7 | -61% | 19 | -2% |
| Fruit compote | 5 | 3 | -42% | 3 | -41% | 3 | -40% | 3 | -27% | 5 | 17% |
| Mixed fruits and olives | 3 | 3 | -8% | 3 | 0% | 4 | 12% | 5 | 31% | 6 | 69% |
| **Potatoes and tubers** | 64 | 73 | 14% | 73 | 15% | 74 | 16% | 75 | 18% | 76 | 20% |
| **Nuts and seeds** |  |  |  |  |  |  |  |  |  |  |  |
| Nuts and seeds, not unsalted | 11 | 12 | 10% | 11 | 6% | 11 | 1% | 10 | -5% | 10 | -6% |
| Nuts and seeds, unsalted | 8 | 10 | 34% | 11 | 51% | 13 | 75% | 15 | 101% | 15 | 104% |
| **Legumes** | 8 | 10 | 36% | 13 | 77% | 15 | 104% | 16 | 109% | 16 | 114% |
|  |  |  |  |  |  |  |  |  |  |  |  |
| **ANIMAL-BASED FOOD GROUPS** |  |  |  |  |  |  |  |  |  |  |  |
| **Dairy** |  |  |  |  |  |  |  |  |  |  |  |
| Milk and yoghurt products, sugar free | 230 | 209 | -9% | 210 | -9% | 210 | -9% | 212 | -8% | 227 | -1% |
| Milk and yoghurt products, sugared | 12 | 7 | -37% | 7 | -41% | 6 | -45% | 5 | -55% | 3 | -72% |
| Dairy, other | 56 | 45 | -19% | 45 | -19% | 46 | -17% | 49 | -11% | 56 | 0% |
| **Meat** |  |  |  |  |  |  |  |  |  |  |  |
| Meat, hot meal, processed | 19 | 17 | -8% | 16 | -11% | 15 | -19% | 11 | -40% | 7 | -64% |
| Meat, cold meal, processed | 19 | 13 | -28% | 13 | -29% | 13 | -31% | 11 | -41% | 7 | -63% |
| Meat beef, unprocessed | 11 | 3 | -73% | 3 | -73% | 3 | -72% | 3 | -70% | 10 | -13% |
| Meat poultry, unprocessed | 17 | 23 | 38% | 23 | 39% | 25 | 47% | 29 | 73% | 33 | 95% |
| Meat pork, unprocessed | 8 | 10 | 20% | 10 | 25% | 11 | 30% | 12 | 49% | 13 | 59% |
| Meat, other | 4 | 1 | -69% | 1 | -67% | 1 | -65% | 2 | -62% | 4 | -11% |
| **Cheese** | 38 | 32 | -17% | 32 | -17% | 32 | -17% | 32 | -17% | 34 | -12% |
| **Fish** |  |  |  |  |  |  |  |  |  |  |  |
| Fish, not fatty | 11 | 7 | -33% | 7 | -32% | 7 | -34% | 7 | -31% | 8 | -29% |
| Fish, fatty | 9 | 19 | 106% | 21 | 123% | 23 | 142% | 23 | 146% | 21 | 120% |
| **Eggs** | 16 | 17 | 6% | 17 | 7% | 18 | 10% | 18 | 14% | 19 | 19% |
| **Dairy and meat replacers** | |  |  |  |  |  |  |  |  |  |  |
| Dairy replacers | 14 | 26 | 86% | 27 | 92% | 26 | 87% | 26 | 85% | 24 | 74% |
| Meat replacers | 7 | 6 | -15% | 6 | -20% | 6 | -22% | 6 | -19% | 7 | -7% |
|  |  |  |  |  |  |  |  |  |  |  |  |
| **MIXED FOOD GROUPS** |  |  |  |  |  |  |  |  |  |  |  |
| **Soups and bouillon** | 25 | 20 | -22% | 20 | -19% | 21 | -17% | 22 | -14% | 21 | -15% |
| **Biscuits and sweet pastries** | 40 | 37 | -8% | 37 | -9% | 37 | -8% | 37 | -8% | 39 | -3% |
| **Contaminants, yeast and sauces** | 36 | 31 | -14% | 31 | -14% | 31 | -13% | 32 | -11% | 33 | -8% |
| **Sugar and confectionary** | |  |  |  |  |  |  |  |  |  |  |
| Sugar, honey, jam, syrup, dessert sauce | 9 | 9 | 0% | 10 | 2% | 10 | 3% | 10 | 2% | 9 | -9% |
| Chocolate, candy bars, chocolate bread toppings | 11 | 11 | -2% | 11 | -3% | 11 | -5% | 11 | -3% | 11 | -3% |
| Sweets without chocolate, other bread toppings | 3 | 5 | 40% | 5 | 40% | 5 | 37% | 4 | 24% | 4 | 31% |
| **Fats and oils** |  |  |  |  |  |  |  |  |  |  |  |
| Fats and oils, soft | 18 | 19 | 5% | 19 | 4% | 19 | 4% | 19 | 6% | 19 | 7% |
| Fats and oils, hard | 5 | 2 | -57% | 2 | -58% | 2 | -60% | 2 | -62% | 2 | -61% |
| **Savoury snacks** |  |  |  |  |  |  |  |  |  |  |  |
| Pretzels, chips, salty biscuits | 8 | 8 | 7% | 8 | 3% | 8 | 0% | 7 | -3% | 7 | -4% |
| Snacks, croquettes, snack rolls | 9 | 12 | 40% | 12 | 37% | 12 | 38% | 12 | 36% | 13 | 48% |
|  |  |  |  |  |  |  |  |  |  |  |  |
| **BEVERAGES** |  |  |  |  |  |  |  |  |  |  |  |
| **Non-alcoholic beverages** | |  |  |  |  |  |  |  |  |  |  |
| Coffee and tea | 940 | 733 | -22% | 753 | -20% | 787 | -16% | 855 | -9% | 948 | 1% |
| Water | 881 | 1149 | 31% | 1127 | 28% | 1096 | 24% | 1043 | 18% | 928 | 5% |
| Soft drinks, sugared | 82 | 50 | -40% | 46 | -44% | 41 | -50% | 35 | -58% | 27 | -67% |
| Soft drinks, sugar free | 61 | 36 | -40% | 36 | -41% | 37 | -39% | 40 | -34% | 65 | 7% |
| Alcohol free beverages | 21 | 15 | -30% | 16 | -25% | 17 | -21% | 18 | -14% | 21 | -2% |
| Juice, fruit | 33 | 10 | -69% | 10 | -71% | 9 | -72% | 9 | -74% | 8 | -75% |
| Juice, vegetables | 1 | 0 | -72% | 0 | -69% | 0 | -66% | 1 | -56% | 2 | 25% |
| **Alcoholic beverages** |  |  |  |  |  |  |  |  |  |  |  |
| Beer | 69 | 83 | 21% | 83 | 21% | 83 | 21% | 84 | 22% | 85 | 23% |
| Wine | 58 | 20 | -66% | 20 | -66% | 21 | -63% | 24 | -58% | 37 | -36% |
| Alcohol, other than beer wine | 5 | 5 | -1% | 5 | -5% | 5 | -4% | 5 | -9% | 5 | -6% |
